# Decoy Receptor 3 Suppresses T-Cell Priming and Promotes Apoptosis of Effector T-Cells in Acute Cell-Mediated Rejection: The Role of Reverse Signaling

**DOI:** 10.3389/fimmu.2022.879648

**Published:** 2022-06-02

**Authors:** Shuo-Chun Weng, Mei-Chin Wen, Shie-Liang Hsieh, Nien-Jung Chen, Der-Cherng Tarng

**Affiliations:** ^1^ Department of Post-Baccalaureate Medicine, College of Medicine, National Chung Hsing University, Taichung, Taiwan; ^2^ Institute of Clinical Medicine, School of Medicine, College of Medicine, National Yang Ming Chiao Tung University, Taipei, Taiwan; ^3^ Center for Geriatrics and Gerontology, Division of Nephrology, Department of Internal Medicine, Taichung Veterans General Hospital, Taichung, Taiwan; ^4^ School of Medicine, Chung Shan Medical University, Taichung, Taiwan; ^5^ Department of Pathology and Laboratory Medicine, Taichung Veterans General Hospital, Taichung, Taiwan; ^6^ Genomics Research Center, Academia Sinica, Taipei, Taiwan; ^7^ Department of Medical Research and Education, Taipei Veterans General Hospital, Taipei, Taiwan; ^8^ Department of Clinical Pharmacy, Taipei Medical University, Taipei, Taiwan; ^9^ Institute of Microbiology and Immunology, College of Life Sciences, National Yang Ming Chiao Tung University, Taipei, Taiwan; ^10^ Inflammation and Immunity Research Center, National Yang Ming Chiao Tung University, Taipei, Taiwan; ^11^ Department and Institute of Physiology, National Yang Ming Chiao Tung University, Taipei, Taiwan; ^12^ Division of Nephrology, Department of Medicine, Taipei Veterans General Hospital, Taipei, Taiwan; ^13^ Center for Intelligent Drug Systems and Smart Bio-devices, National Yang Ming Chiao Tung University, Hsinchu, Taiwan

**Keywords:** adaptor protein, decoy receptor, minor antigenic determinant, knockout, phenotype, transgenic mice

## Abstract

**Background:**

Decoy receptor 3 (DcR3) belongs to the tumor necrosis factor (TNF) receptor superfamily and neutralizes TNF ligands, including FasL and TRAIL, to prevent T activation during T-cell priming. However, the cellular mechanisms underlying acute cell-mediated rejection (ACMR) remain unknown.

**Methods:**

We generated DcR3 transgenic (Tg) mice and mice with high DcR3 expression (HDE) to study both *in vivo* and *in vitro*. FasR RNA knockdown in immortalized CD4^+^CD8^+^ T-cells was used to survey the role of DcR3 on FasR/Fas-associated protein with death domain (FADD)/caspase 8 pathway and its cross-link to TNF receptor-associated factor 1 (TNFR1)-associated death domain protein (TRADD) in suppressing TNFR1. TNF/TRADD knockout mice were used to show the importance of TNF adaptor protein.

**Results:**

DcR3.Fc suppressed C57BL/6 female T-cell activation and transformation into CD4^+^CD69^+^, CD4^+^CD44^+^, and CD4^+^CD25^+^Foxp3^+^ when compared with isotype IgG1 and its co-treatment with FasL/TRAIL after exposing to bone marrow-derived dendritic cells (BMDCs) that carried alloantigen with male H-Y and minor antigenic determinant. Interleukin-17 and interferon-γ productions by BMDC-activated T-cells were lowered after co-treating with DcR3.Fc. DcR3.Fc induced effector T-cells (Teffs) and was susceptible to FasR-mediated apoptosis through the FADD/TRADD/caspase 8 pathway. After exposing to DcR3.Fc, TRADD was silenced, likely turning down the inflammatory response. The systemic effects of DcR3 Tg mice and HDE phenotype induced by the promoter of cytomegalovirus not only attenuated ACMR severity but also ameliorated the high serum creatinine and blood urea nitrogen levels even with high T-cell exposure frequencies. Besides this, DcR3 has minor biological effects on both MHC-matched and MHC-mismatched models.

**Conclusions:**

High DcR3 doses protect renal tubular epithelial cells from acute T-cell attack during the T-cell priming stage *via* interfering with TNF ligand-mediated reverse signaling and possibly promoting Teff apoptosis through FasR upregulation. Our findings supported that the decoy receptor is involved in T-cell modulation in kidney transplant rejection.

## Introduction

Decoy receptor 3 (DcR3) protects transplanted islet cells, skin allograft, and the heart mainly through an interaction with tumor necrosis factor (TNF) family ligands, such as FasL and LIGHT (1–3). DcR3, a soluble TNF receptor superfamily 6B (TR6) member, has a pro-tolerogenic potential on the immune system and immune-evasive function. It induces CD14^+^ monocyte differentiation into CD1a^low^CD40^low^CD54^low^CD80^low^CD86^high^ dendritic cells (DCs) and skews the differentiation of T-cells into the Th2 phenotype (4–6). We have previously reported that a soluble receptor activator of NF-kB (RANK).Fc fusion protein (a member of the TNFR superfamily) dramatically suppresses interferon (IFN)-γ secretion when T-cells were primed in conditions favoring Th1 differentiation (7). DcR3 also inhibits anti-CD3 and LIGHT-induced T-cell actin polymerization, pseudopodia formation, and T-cell aggregation (8). Local transgenic DcR3 expression in pancreatic beta cells does not affect systemic lymphocyte properties or Th1 or Treg cell development (9); however, its systemic expression in HNT-DcR3 double transgenic mice or with plasmid human DcR3 gene delivery produces a Th2 cell-biased phenotype (6, 10). However, regarding the development of intragraft ratio of effector T (Teffs) and regulatory T (Tregs) during T-cell induction, the role of DcR3 in determining graft kidney survival has not been elucidated.

Both FasL/FasR and TRAIL/TRAIL-R systems are involved in activation-induced T-cell death, through which T-cells commit fratricide or suicide to terminate an auto-immune response (11). Although FasL and TRAIL are death-inducing TNF family members in FasL- or TRAIL-sensitive transformed cells, they also co-stimulate T-cells in conjunction with sub-optimal anti-CD3 (12). In the skin allograft animal model, FasL and TRAIL, which are differently and constitutively expressed by activated Treg cells, regulate Teff suppression mediated by Treg *via* apoptosis induction both *in vitro* and *in vivo* (13). FasL and TRAIL also play an important role in CD4^+^ and CD8^+^ cytotoxic reactions during xenotransplantation in human peripheral blood mononuclear cells and swine endothelial cells. Their action is blocked by the overexpression of decoy Fas, prolonging the allograft survival.

We have previously reported that endogenous DcR3, locally produced by mononuclear cells and renal tubular epithelial cells (RTECs), is positively correlated with both T-cell mediated rejection (TCMR) severity and adverse allograft survival in human renal transplant recipients (RTRs) (14). Therefore, we tested the hypothesis that DcR3 critically neutralizes FasL/TRAIL-dependent T-cell priming, decreases TNFR1 inflammatory cytokines, and upregulates FasR downstream pathway to induce T-cell apoptosis. Such mechanisms are thereby involved in allograft protection pathogenesis. To elucidate the protective signal pathways emanating from DcR3, we have established both *in vitro* and *in vivo* ACMR models to study the underlying mechanisms controlling immune tolerance.

## Materials and Methods

### Antibodies and Reagents

For immunohistochemical (IHC) staining, monoclonal antibodies (mAbs) were used against cleaved caspase 3, which was followed by the same procedure with LIGHT and CTLA4. Anti-FasL, anti-LIGHT, anti-TRAIL, and anti-TL1A antibodies were used for western blot. The antibodies used for immunofluorescence (IF) double staining were anti-CD45, CD3, CD4, and CD8 Ab for T-cell markers, anti-CD20 Ab for B-cell marker, and anti-CD68 Ab for macrophage. For DcR3 Ab, the incubation duration was overnight at 4°C. Immunoreactivity was detected with fluorochrome/secondary antibodies, including anti-goat (Alexa Fluor, wavelength = 647 nm, Abcam) and anti-rabbit (Alexa Fluor, wavelength = 488 nm, Abcam) IgG for DcR3 and CD markers, respectively. We used 4′-6-diamidino-2-phenylindole to bind with double-strand DNA in the nucleus, aided under 358 nm (blue light). An imaging technique was used to increase the optical resolution and contrast of micrographs from confocal microscopy (Olympus FV1000, Japan). The reagents for T-cell study included FasL (Abcam), TRAIL (Sino Biological), DcR3.Fc (Sino Biological), and human IgG1 alone (Millipore). The mAbs for flow cytometry included CD4, CD8, CD25, Foxp3, CD44, CD69, FasL, FasR, TRAIL-R2, TNFR1, and TNFR2 (**Supplementary Table S1**).

### *In Vitro* Co-Incubation of Male DCs and Female T-Cells

Bone marrow-derived dendritic cells (BMDCs) from 5–7-week-old male C57BL/6 mice were cultured in a petri dish with 6 ml Roswell Park Memorial Institute (RPMI) and 20 ng/ml granulocyte–macrophage colony-stimulating factor (GM-CSF, ProSpec-Tany TechnoGene Ltd., Ness Ziona, Israel). RPMI and GM-CSF were changed on days 3, 6, and 8 while culturing BMDCs. On day 9, 100 ng/ml lipopolysaccharide (LPS, collected from *Escherichia coli* 0111: B4; r-irradiated, BioXtra) was used to stimulate the BMDCs for 24 h. Initially, RBC was lysed using the ACK lysing buffer (Affymetrix, eBioscience), and the presence of mature BMDC was confirmed using positive staining with MHCII (eBioscience) and CD11c-PE (eBioscience) (**Supplementary Figure S1A**).

CD4^+^ and CD8^+^ T-cells were purified from the total lymphocytes using high-gradient magnetic sorting based on the VARIOMACS technique with anti-CD4^+^ and CD8^+^ microbeads (Miltenyi Biotec Inc., USA). Fluorescence-activated cell sorter-sorted CD4^+^/CD8^+^ T-cells were obtained from female C57BL/6 lymph nodes (LNs) and spleen (15). Then, female LN cells were sensitized with male BMDCs containing alloantigen and Y-chromosome for 3 days (16, 17). Flow cytometry (BD Bioscience) with carboxyfluorescein diacetate succinimidyl ester (CFSE, Invitrogen) and GFP gene-carrying mice were used to assess T-cell proliferation. We divided T-cell proliferation into four groups: T-cells alone, BMDCs with T-cells, BMDCs with T-cells and 10 ng/ml rIL-2 (R&D Systems), and BMDCs with T-cells with 10 ng/ml rIL-2 and 5 μg/ml anti-CD3 (eBioscience) (**Supplementary Figure S1A**). All aliquots were labeled with CFSE.

### Mice

We used male CD68 promoter-driven DcR3-transgenic (CD68-DcR3 Tg, C57BL/6 background) mice, B6 TNF/TNF receptor-associated factor 1 (TNFR1)-associated death domain protein (TRADD) double knockout (DKO) mice, and B6 GFP knock-in mice supplied by Dr. NJ Chen (gifted by Dr. SL Hsieh, Genomics Research Center, Academia Sinica, Taipei, Taiwan). The animals were maintained on a standard mouse chow diet, adequate room temperature, humidity, and light/dark cycle. The B6 DcR3 Tg mice were characterized by a 4.7-kb CD68-DcR3 cassette annealed to the CD68 promoter (4, 18), and DcR3 was specifically expressed on macrophages. To evaluate the immune modulation of DcR3 on mice with acute cellular rejection (ACR), we designed four experimental mice groups (*n* = 5 per group): wild-type (WT) mice, DcR3 Tg mice, WT mice with empty vector, and WT mice with hDcR3 plasmid injection (**Supplementary Figure S1B**). All experiments were conducted with age-matched (7–8 weeks old) mice housed under specific-pathogen-free conditions. The ACR model was induced in a two-step procedure.

### *In Vitro* and *In Vivo* ACR Animal Model

The *in vitro* ACR co-culture system included naïve or activated primary T-cells from female mice and RTECs from male mice together with male BMDCs, GM-CSF, and rIL-2, and those distinct patterns in Teff and Treg cells upon activation with TCR co-stimulation.

To facilitate heterotopic transplantation and allograft rejection, the costal and hypochondriac regions from both sides of another 6–10-week-old male mouse were used for surgery. We created a tunnel/pouch in the renal cortex in the male recipient B6 mouse and injected 1–3 × 10^6^ activated CD4^+^/CD8^+^ female T-cells with amplified H-Y memory (**Supplementary Figure S1C**). This invention is granted under the law by the United States Patent and Trademark Office (Patent US 10,835,622 B2, November 17, 2020). To strengthen the ACR mice model, concurrent BMDCs and T-cells were transferred to the renal cortex of male mice (19, 20). Using this model, we evaluated tubular injury and interstitial inflammation based on the Banff classification (21). Acute allograft injury was confirmed by the area destroyed in both kidneys and the serum renal function of a given mouse.

### Hydrodynamics-Based Gene Delivery

The human DcR3 (hDcR3) plasmid was prepared from *E. coli* growing on the LB shock culture medium. DH5alpha/pcDNA3_hDcR3_IgGFc or DH5alpha/pcDNA3_IgGFc was isolated with the Geneaid™ plasmid Maxi kit, and potential endotoxins were removed from this promoter of cytomegalovirus (pCMV) vector (Clontech, Palo Alto, CA, USA) with EndotoxinOUT™ Resin (catalog #786-367). The expression constructs or the empty vector was administered to the B6 WT mice through the tail vein *via* hydrodynamics-based gene delivery using a single dose of 50 μg/10 g body hDcR3 plasmid (*n* = 17) (10, 22). The animals were then euthanized between days 7 and 28 after plasmid administration, and serum and kidney tissues were stored until analysis. Typically, the serum DcR3 levels increased on day 1, peaked on day 5, declined gradually to day 14, and returned to baseline by day 28, as demonstrated using ELISA. Meanwhile, we also injected the mouse renal cortex with activated T-cells on day 0 to create an ACR model.

### Interpreting High-Expression (pCMV-hDcR3^high^) or Low-Expression (pCMV-hDcR3^low^) Phenotype

WT mice transfected with hDcR3 plasmid with high (pCMV-hDcR3^high^) or low (pCMV-hDcR3^low^) DcR3 expression phenotype regulated the disease. High and low DcR3 expression was defined by serum DcR3 protein levels above and below the median level (37.0 ng/ml), respectively. The interquartile range was 10.5–66.5 ng/ml.

### Study of Primary T-Cells and Immortalized Mouse T-Cells

Primary CD4^+^/CD8^+^ T-cells were isolated from WT B6 mice. Female T-cells and activated male BMDCs were co-cultured for 18 to 120 h with rIL-2. The aliquots were treated with IgG1 or DcR3.Fc (10 µg/ml) with or without 100 ng/ml FasL (23). The other groups were treated separately with IgG1 or DcR3.Fc with or without 100 ng/ml TRAIL (24). For flow cytometry, antibodies with fixable and viable dyes were applied on the BD Bioscience machine, and T-cell phenotypes were analyzed with Cytobank (Cytobank; Santa Clara, CA, USA) or FlowJo software (Becton Dickinson; Franklin Lakes, NJ, USA). The purity of lymphocytes was determined based on CD4 and CD8. Immortalized mouse CD4^+^CD8^+^ T-cells (MOHITO) (25) with Balb/c origin and IL-7 and IL-2 dependence were used to study the underlying mechanisms.

### Cell Proliferation Assay

To determine the effects of DcR3 on T-cell priming, female CD4^+^/CD8^+^ T-cells were incubated with CFSE and then co-cultured with male BMDCs, followed by addition of various reagents for 36 h. Proliferation was evaluated by flow cytometry with CFSE dye incorporation.

### Apoptosis Analysis by TUNEL Assay and Annexin-V-FITC/PI Staining

For the transferase dUTP nick-end labeling (TUNEL) assay, an *in situ* cell death detection kit (Vazyme) was used according to the manufacturer’s instructions. For each sample, TUNEL-positive tubular or T-cells were counted in 10 non-overlapping fields. Before and after DcR3.Fc treatment, the cells were double-stained with annexin-V-FITC and propidium iodide (PI; BD Pharmingen™).

### Primary RTEC Culture

Kidneys were harvested from neonate male 2-month-old 18–22 g B6 mice using sterile procedures. One 6-well plate with phosphate-buffered saline + penicillin + streptomycin at 0.5 ml (Gibco, Life Technologies; Paisley, UK) was used to wash the harvested kidneys. Then, the native renal capsule and renal hilum were removed, and the renal cortices were dissected visually into approximately 1- to 2-mm^2^-wide pieces and digested with 100 μl collagenase. After digestion, the supernatant was sieved through two nylon meshes (100 and 75 μm pore size, Fisher Scientific). Only the 75–80-μm sieves yielded a large number of proximal tubules. A 2-ml suspension containing Percoll 45% (Sigma-Aldrich) and a cell suspension in 1:1 proportion were centrifuged at 4°C and 13,000 × *g* for 30 min. The bottom layer was then retrieved (26).

### Electroporation for the Transmission of FasR shRNA

The endogenous protein was knocked down by transiently transfecting the cell lines with shRNA oligonucleotides. FasR shRNA (shFasR) and pLKO_TRC001 control (both from RNAi core, Academia Sinica; Taipei, Taiwan) were electroporated into MOHITO cells using the Neon™ Electroporation Transfection system (100-µl transfection volume, 1 × 10^6^–5 × 10^6^ T-cells, and 6-well, 60-mm, or 10-cm culture dish).

### Western Blot Analysis and Reverse Transcription-Polymerase Chain Reaction of Cell and Kidney Lysates

Proteins in cells or kidney lysates were separated electrophoretically on Tris-glycine SDS-polyacrylamide gel and detected by western blot analysis according to standard protocols. The proteins were then electro-blotted on a nitrocellulose transfer membrane and incubated overnight at 4°C using rabbit horseradish peroxidase (HRP)-conjugated (polyclonal) antibodies against target proteins and mouse HRP-conjugated (monoclonal) antibodies against beta-actin and GAPDH. Finally, the blot was subjected to chemiluminescent detection according to the manufacturer’s instructions. The gel analysis and quantitative values were obtained using Gene Tools (version 08-3d.3., SynGene; Cambridge, UK).

For real-time polymerase chain reaction (qPCR), the total RNA (cells and kidney lysates) was purified using the TRIzol reagent according to the manufacturer’s instructions. The qPCR analyses were carried out using StepOnePlus™ Real-Time PCR System (ABI) and Power SYBR Green PCR Master Mix (Thermo Fisher Scientific) as a qPCR reagent kit. The analyses were carried out using a Lightcycler system (Roche), with GAPDH RNA serving as an internal control. The qPCRs were performed to detect RNA expressions in mice and humans (**Supplementary Tables S2, S3**).

### Statistical Analyses

The *in vitro* and *in vivo* results were expressed as the mean ± standard deviation (SD) or standard error (SE). The results from multiple groups were analyzed using one-way analysis of variance (ANOVA). We used Kruskal–Wallis test followed by Bonferroni *post-hoc* analysis for multiple testing. For two independent groups, nonparametric Mann–Whitney *U*-test was used. The percentages of infiltration in gross view of mouse kidneys were quantified with the software Image-Pro Plus 6.0 (Media Cybernetics; Silver Spring, MD, USA). Analysis of the 3,3′-diaminobenzidine-positive area was carried out using Image J (version 1.16). Dot plots and graphs were generated with the software GraphPad v5. Receiver operating characteristic curves were compared using the MedCalc software (1993–2008, Frank Schoonjans, The Netherlands).

## Results

### DcR3 Significantly Attenuates Tubulitis and Interstitial Inflammation and Prevents Renal Function Deterioration in ACR Models

**Figure 1:**
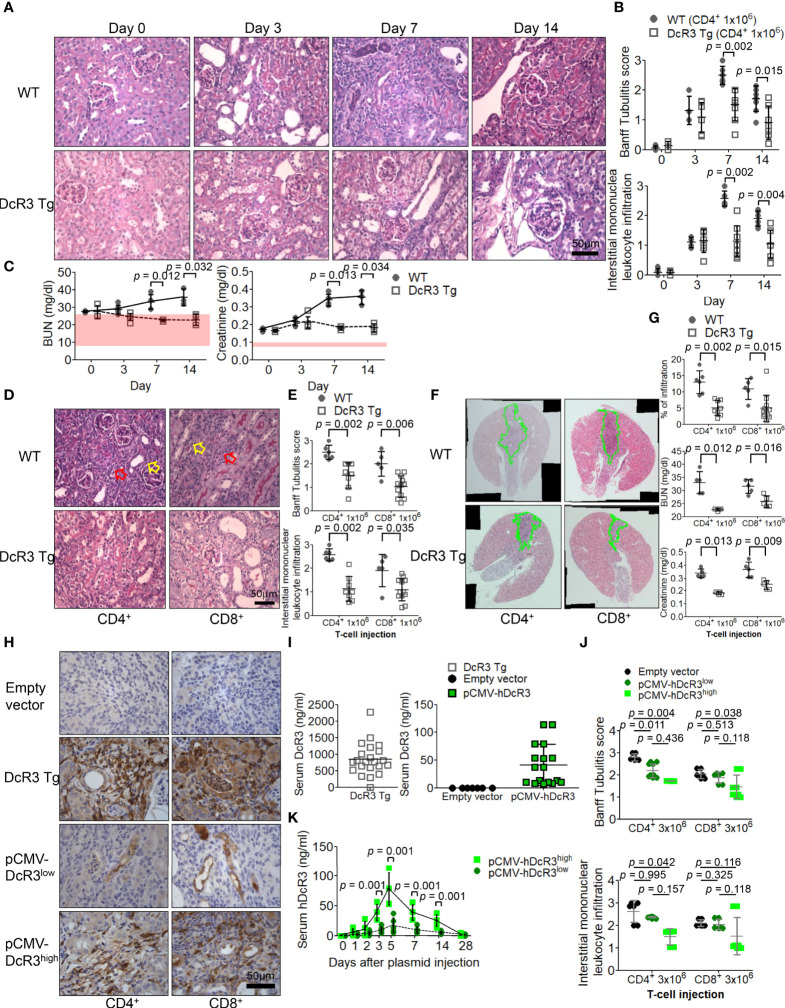
Decoy receptor 3 (DcR3) ameliorated renal dysfunction in the acute cell rejection model. **(A)** Representative periodic acid–Schiff staining of wild-type (WT; upper panel) and DcR3 transgenic (Tg; lower panel) mice kidneys (×400). **(B)** Banff tubulitis (t) and interstitial inflammation (i) scores. **(C)** Serum blood urea nitrogen (BUN) and creatinine levels were measured at days 0, 3, 7, and 14 post-injection (5 mice per group; the normal ranges of serum BUN and creatinine levels are indicated by the light pink rectangles). **(D)** Histopathology of WT and DcR3 Tg mice at day 7 after 1 × 10^6^ CD4^+^ and CD8^+^ T-cell injection (hollow yellowish and red arrows highlight tubulitis and interstitial mononuclear leukocyte infiltration, respectively; ×400). **(E)** Banff t and i scores at day 7 after 1 × 10^6^ CD4^+^ and CD8^+^ T-cell injection. **(F)** Infiltrated area from two sagittal sections of each kidney stained with hematoxylin and eosin at day 7. **(G)** Percentage of infiltrated area, serum BUN, and creatinine of WT and DcR3 Tg mice at day 7 after 1 × 10^6^ CD4^+^ and CD8^+^ T-cell injection. **(H)** DcR3 immunohistochemical staining of plasmid tail vein injection mice in kidney rejection model (×400). **(I)** Serum DcR3 levels in Tg, empty vector, and pCMV-hDcR3-delivered WT mice. **(J)** Banff t and i scores in mice treated with empty vector and human DcR3 plasmid at day 7 after 3 × 10^6^ CD4^+^ and CD8^+^ T-cell injection. **(K)** WT mice transferred hDcR3 plasmid with high expression (pCMV-hDcR3^high^) or low expression (pCMV-hDcR3^low^) of DcR3 phenotype to regulate the disease. Both the high and low DcR3 expressions were defined by median DcR3 protein levels in the serum. **(J)** The results of multiple groups were analyzed using Kruskal–Wallis test, followed by Bonferroni *post-hoc* analysis, and those data were presented as mean ± SD.

Compared with that in sham control of WT mice on day 0, tubulitis and interstitial mononuclear cell infiltration of bilateral kidneys were the strongest after alloantigen exposure in ACR WT mice on day 7 (**Figures 1A, B**). Compared with WT mice on days 7 and 14, DcR3 Tg mice ameliorated tubulitis and interstitial inflammation (**Figures 1A, B**) and showed lesser deterioration of renal function (**Figure 1C**). DcR3 reduced not only the activated CD4^+^/CD8^+^-induced inflammatory response (**Figures 1D, E**) but also the destruction area (**Figure 1F**) and renal dysfunction progression (**Figure 1G**). However, DcR3 Tg mice expressed 780.0 ng/ml protein (interquartile range, 579.5–1,128.0 ng/ml; **Figures 1H, I**) which was higher than the physiological range in patients showing kidney allograft rejection (serum DcR3 levels: 0–11.25 ng/ml in patients with ACR) (14).

Activated CD4^+^/CD8^+^ induction appeared both dose dependent (3 × 10^6^ *versus* 1 × 10^6^) and time dependent. Such changes gradually diminished in DcR3 Tg mice (**Supplementary Figure S2**) and pCMV-hDcR3-treated WT mice (**Figure 1J**). Moreover, the DcR3 protein levels rapidly increased on day 5 and then declined back to baseline by day 28 after a single DcR3 plasmid injection (**Figure 1K**). WT mice transfected with hDcR3 plasmid with high-expression (pCMV-hDcR3^high^) or low-expression (pCMV-hDcR3^low^) phenotype regulated the disease after a high-dose (3 × 10^6^) T-cell injection, reducing tissue infiltration (**Figure 1J**) and slowing renal progression (**Supplementary Figure S3**) than the control mice treated with empty vector.

### DcR3 Participates in T-Cell Immunobiology in Line With Suppressing the Corresponding Ligands, Inducing Anti-Inflammatory Cytokines and Death Decoy Function

**Figure 2:**
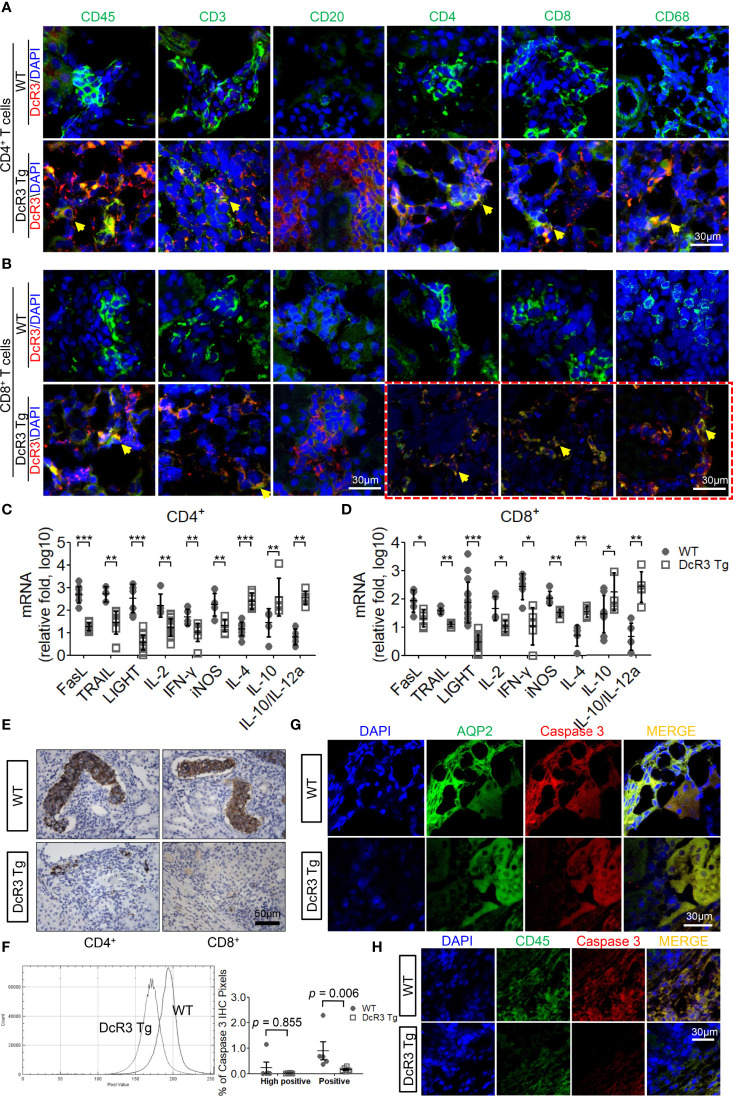
The decoy receptor 3 (DcR3) interaction with T-cell markers correlated with the interference of corresponding ligands, inducing anti-inflammatory cytokines and death decoy function. **(A, B)** Merged immunofluorescence (IF) double staining for CD45, CD3, CD20, CD4, CD8, CD68, and DcR3 in acute cell rejection (ACR) wild-type (WT) and DcR3 transgenic (Tg) mice (×600). CD4, CD8, CD68, and DcR3 IF double staining after CD8^+^ T-cell injection (×1,200). **(C, D)** Real-time PCR analysis of mRNA levels of the indicated genes in the kidney tissue of ACR WT or DcR3 Tg mice at day 7 after activated CD4^+^ and CD8^+^ T-cell injection. Data are presented as fold change relative to the GAPDH mRNA level and are representative of at least five independent experiments, followed by a Mann–Whitney *U*-test. Dot plots represent mean ± SD. **(E)** Cleaved caspase 3 immunohistochemical (IHC) staining of CD4^+^- or CD8^+^-related ACR WT and DcR3 Tg mice at day 7. **(F)** Quantitative data of cleaved caspase 3 IHC staining. The percentage of high positive and positive caspase 3 IHC staining of kidney tissue, respectively, were examined and averaged under 20 randomly selected high-power fields [×200; WT (*n* = 5) and DcR3 Tg (*n* = 6)]. **(G, H)** IF double staining was performed for cleaved caspase 3, aquaporin 2, and CD45 from WT and DcR3 Tg mice. *p < 0.05, **p < 0.01, and ***p < 0.001 compared with WT.

We examined the massive infiltration of immune cells to detect whether DcR3 regulates immune cells in TCMR. We also found the co-localization of CD45 and DcR3 as well as T-cell markers (CD3, CD4, and CD8) and DcR3 in areas proximal to the plasma membrane of inflamed cells of ACR DcR3 Tg mice. A similar CD68 and DcR3 co-localization was found in ACR DcR3 Tg mice after CD4^+^ or CD8^+^ T-cell injection (**Figures 2A, B** and **Supplementary Figure S4**). As expected, marked decreases were found based on IF staining with leukocytes, lymphocytes, and macrophages in ACR DcR3 Tg than that in ACR WT. Consistent with the results in both CD4^+^/CD8^+^-induced ACR WT and ACR DcR3 Tg mice, we did not detect the presence of CD20 T-cells. Since mouse and rat genomes do not have DcR3, assessing DcR3 expression with immune cell surface markers based on green fluorescence appearing around the membrane area is not feasible (**Figures 2A, B**). In addition, the ACR model was further confirmed by the activation of DcR3-regulated apoptosis pathway, allograft rejection signaling, TCMR transcripts (**Supplementary Figures S5A, B**), and immune regulation (**Supplementary Figure S5C**) determined by the mRNA microarray of kidney lysates from National Health Research Institutes Microarray Core facility.

On day 7 after ACR, the FasL, TRAIL, and LIGHT mRNA expressions in DcR3 Tg mice were significantly suppressed. Moreover, the pro-inflammatory cytokines (IL-2, IFN-γ, and iNOS) were downregulated and the anti-inflammatory cytokines (IL-4, IL-10, IL10/IL-12a) were upregulated in DcR3 Tg mice than those in WT mice (**Figures 2C, D**).

Anti-apoptosis is a signature feature of DcR3-mediated allograft protection (1, 2). To further explore the apoptosis-related immune regulation of DcR3 on mononuclear cells and RTECs, we studied whether caspase 3 is associated with DcR3 functioning (27). It is interesting to note that strong active caspase 3 staining was found on damaged tubules surrounded by mononuclear cells in ACR WT mice, whereas such staining was weak in the ACR DcR3 Tg mice (**Figure 2E**). Besides this, in the quantitative data, we could see the smaller percentage of high positive and positive areas of caspase 3 IHC staining and pixel values in DcR3 Tg mice than that in WT mice (**Figure 2F**). Among the major differences in tissue IF double staining, cleaved caspase 3 was separately upregulated in injured tubuli (AQP2) in ACR WT mice (**Figure 2G**) and infiltrated monocytes (CD45) in ACR DcR3 Tg mice (**Figure 2H**). On day 7 after GFP T-cell injection, damaged tubuli were notably destroyed concurrently by major recipient-derived T-cells and a small amount of donor-derived activated T-cells of ACR mice (**Supplementary Figure S6**).

### DcR3 Potentially Protects RTECs From Acute T-Cell Attack During the Priming Stage

**Figure 3:**
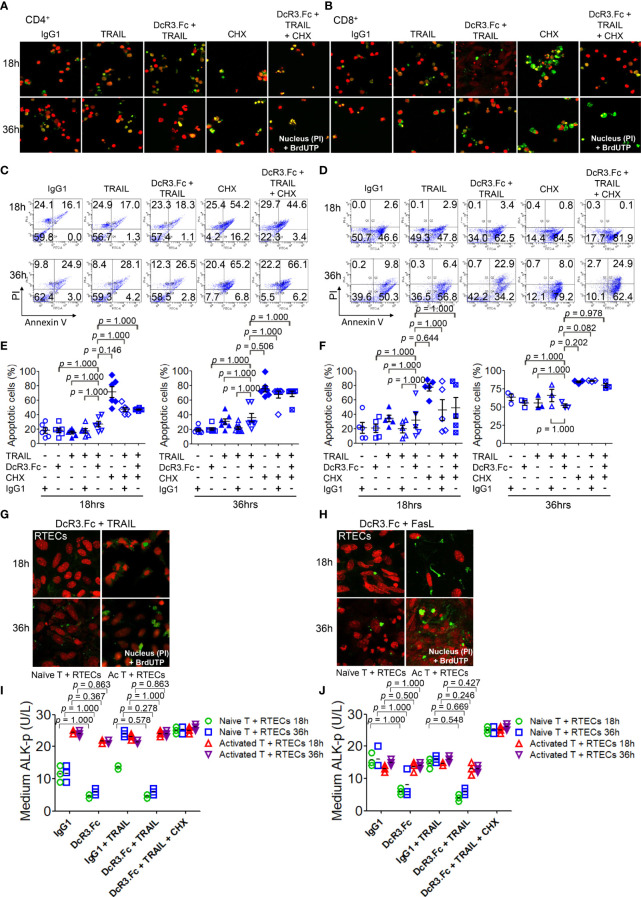
Decoy receptor 3 protects renal tubular epithelial cells (RTECs) from T-cell attack during the priming stage *in vitro*. **(A, B)** Terminal deoxynucleotidyl transferase dUTP nick-end labeling (TUNEL) assay of activated CD4^+^ and CD8^+^ T-cells, respectively. **(C, D)** Flow cytometry with Annexin V/PI staining. **(E, F)** Quantitative data of apoptotic cells of activated CD4^+^ (IL-2 and anti-CD3)/CD8^+^ (IL-2 and anti-CD3 and anti-CD28) T-cells were evaluated by the Kruskal–Wallis test, followed by Bonferroni *post-hoc* analysis for multiple testing. **(E, F)** are part of a summary of **(C, D)**. **(G, H)** TUNEL assay of RTECs co-cultured with naïve or activated CD4^+^/CD8^+^ T-cells. **(I, J)** Medium alkaline phosphatase (ALK-p) of the RTECs co-cultured with naïve or activated CD4^+^ or CD8^+^ T-cells, respectively, was analyzed by Kruskal–Wallis test, followed by Bonferroni *post-hoc* analysis, and those data were presented as mean ± SD. One set of the representative data from at least three experiments (*n* = 3) is shown. The group with cycloheximide was a positive control of apoptosis. IgG1, human IgG1; CHX, cycloheximide; naïve T, from female LN cells or spleen; activated T, female naïve T-cells activated by male bone marrow-derived dendritic cells + lipopolysaccharide.

DcR3 enhances apoptosis in TRAIL-sensitive transformed T-cells without triggering any apoptotic event (28). TRAIL and FasL, which play major roles in allograft rejection (13, 29), were detected in T-cells co-cultured with BMDCs using western blot (**Supplementary Figure S7**). We investigated whether DcR3 changed the apoptosis of activated CD4^+^/CD8^+^ T-cells *in vitro*. The apoptotic morphology (**Figures 3A, B**) and flow cytometry analysis (**Figures 3C, D**) for activated CD4^+^/CD8^+^ T-cells appeared to be similar among IgG1, DcR3.Fc, TRAIL alone, and DcR3.Fc+TRAIL at 18 or 36 h (**Figures 3E, F**). **Figures 3E, F** are part of the summary in **Figures 3C, D**.

We also examined the effects of DcR3 on RTECs co-cultured with naïve T-cells (from female LN cells or spleen) or activated T-cells (female naïve T-cells activated by male BMDCs + LPS). As expected, we potentially found protective effects of DcR3 on RTECs after adding DcR3.Fc alone or DcR3.Fc with FasL or TRAIL using the TUNEL assay (**Figures 3G, H**) and medium ALK-p (**Figures 3I, J**) (30) than those of the IgG1 control. The western blot results also showed the protective effect of DcR3.Fc on RTECs co-cultured with naïve T-cells (**Supplementary Figure S8**). These observations *in vitro* led to the hypothesis that DcR3 is likely involved in T-cell priming and thereby contributes to protecting ACR both *in vitro* and *in vivo*.

### DcR3 Critically Suppresses Teff Activation/Proliferation *Via* Interfering With TNF Ligand-Mediated Reverse Signaling

**Figure 4:**
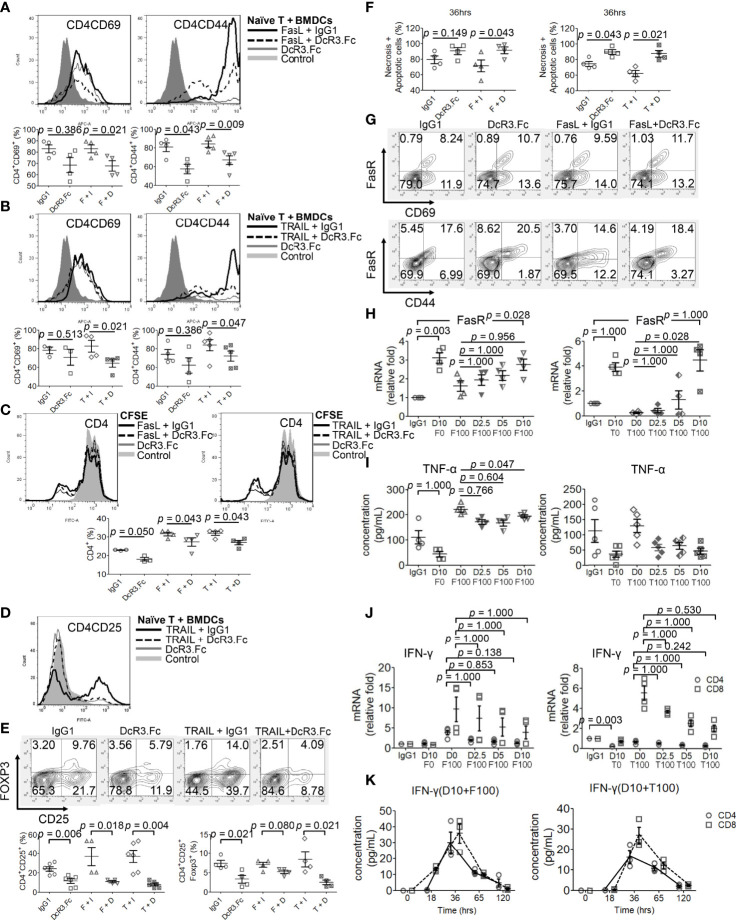
Decoy receptor 3 (DcR3) suppresses FasL/TRAIL-related T-cell activation and proliferation, promotes effector T-cell (Teff) necrosis and apoptosis, and inhibits pro-inflammatory cytokines. **(A, B)** Teffs are obtained from naïve female T-cells from LN cells or spleen and are activated by male bone marrow-derived dendritic cells + lipopolysaccharide. IgG1, DcR3.Fc, FasL/TRAIL + IgG1, and FasL/TRAIL + DcR3.Fc-treated wild-type (WT) T-cells for 24 h were subcategorized into CD4CD69 and CD4CD44 and further presented by histogram and percentage of the indicated immune cells. The cells were double-stained with anti-mCD4-allophycocyanin (APC)-cyanine7(cy7)/CD44-APC or CD69-APC. Control means without treatment, not even IgG1 alone. **(C)** Carboxyfluorescein diacetate, succinimidyl ester (CFSE) labeling assay. CFSE-labeled T-cells were treated with IgG1, DcR3.Fc, FasL/TRAIL + IgG1, or FasL/TRAIL + DcR3.Fc for 36 h before the flow cytometry analysis. Control means without treatment, not even IgG1 alone. **(D, E)** Regulatory T-cells were triple-stained with anti-mCD4-APC-cy7/CD25-FITC/Foxp3-Alexa Fluor and subjected to flow cytometry; the percentage of positively stained cells is indicated in each quadrant plot. Control means without treatment, not even IgG1 alone. **(F)** Necrotic and apoptotic cells were evaluated by Annexin-V-FITC/PI staining. Left panel, CD4; right panel, CD4. **(G)** Detection of surface FasR in the indicated Teffs. **(H)** Dose-dependent effect of DcR3.Fc on FasR expression in the indicated Teffs. The cells were incubated with 100 ng/ml FasL, 100 ng/ml TRAIL, and 0, 2.5, 5, and 10 μg/ml DcR3.Fc, and then the FasR mRNA levels were determined using real-time PCR. **(I)** Tumor necrosis factor (TNF)-α secretion suppression. The supernatants were harvested 36 h after T-cell priming to test TNF-α using ELISA. **(J)** Interferon (IFN)-γ inhibition in DcR3.Fc-treated Teffs was evaluated using real-time PCR. **(K)** IFN-γ of culture medium in time sequence (solid lines, CD4 T-cells; dashed lines, CD8 T-cells). **(A–K)** All experiment systems are *in vitro* systems of acute cell rejection model for 36 h. One set of representative data from at least four experiments (n=4) is shown. The Kruskal–Wallis test, followed by Bonferroni *post-hoc* analysis, was used for multiple testing **(H–J)**. For two independent groups **(A–C, E, F)**, a nonparametric test, Mann–Whitney *U*-test, was used. The data are presented as mean ± SE. F, FasL; I, human IgG1; D, DcR3.Fc; T, TRAIL; D0, 0 μg/ml DcR3.Fc; D2.5, 2.5 μg/ml DcR3.Fc; D5, 5 μg/ml DcR3.Fc; D10, 10 μg/ml DcR3.Fc; F0, 0 ng/ml FasL; F100, 100 ng/ml FasL; T100, 100 ng/ml TRAIL.

Naïve female CD4^+^/CD8^+^ T-cells were incubated with activated male BMDCs under different reagents for 24 h, and the T-cell phenotype was examined. DcR3 was critical for CD4^+^CD69^hi^, CD4^+^CD44^hi^ (**Figures 4A, B**), CD8^+^CD69^hi^, and CD8^+^CD44^hi^ T-cell induction (**Supplementary Figures S9A, B**). CD4^+^CD69^+^ and CD4^+^CD44^+^ cells, in particular, are prominently induced with FasL + IgG1 (82.9 ± 7.1 and 84.2 ± 7.6%, respectively) and TRAIL + IgG1 (82.8 ± 12.4 and 84.1 ± 13.4%, respectively) but are less induced with FasL + DcR3.Fc (65.1 ± 7.0 and 67.1 ± 10.3%, respectively) and TRAIL + DcR3.Fc (64.6 ± 8.8 and 72.2 ± 12.0%, respectively) (**Figures 4A, B**). In addition, the cell proliferation assay with CFSE also showed that DcR3 suppressed CD4^+^/CD8^+^ T-cell priming through FasL/TRAIL involvement (**Figure 4C** and **Supplementary Figure S9C**). DcR3 also induced CD4^+^CD25^+^Foxp3^lo^ (**Figures 4D, E**) and CD8^+^CD25^+^Foxp3^lo^ (**Supplementary Figures S9D, E**) under a representative gating strategy (**Supplementary Figure S10**). However, CD4^+^CD25^+^Foxp3^+^ cells are rarely induced with DcR3.Fc alone (3.3 ± 2.0%) than that with IgG1 control (7.4 ± 1.6%) or with TRAIL + DcR3.Fc (2.5 ± 1.2%) than that with TRAIL + IgG1 (8.5 ± 3.9%) (**Figures 4D, E**).

### DcR3 Might Suppress the Mouse T-Cell-Mediated Immune Responses of MHC-Matched and MHC-Mismatched Models

Considering the different frequencies of male antigen-specific naïve T-cells from MHC-mismatched female mice or rats, we performed the stimulated proliferation of CD4 T-cells under the MHC disparity and found biologically minor impacts of DcR3 (**Supplementary Figures S11D, E**). However, mouse or rat RTECs did not express class II MHC without or with exogenous IFN-γ (**Supplementary Figures S11A–C**).

### DcR3 May Promote Teff Apoptosis Through FasR Upregulation Prone to Anti-Th1 Phenotype

However, DcR3.Fc unexpectedly and strongly induced Teff apoptosis (**Figure 4F**). Upregulating FasR expression by DcR3.Fc was mildly detected by flow cytometry at 36 h (**Figure 4G**). **Figure 4G** shows that FasR increased from 8.2 to 10.7% (CD69 cells) and from 17.6 to 20.5% (CD44 cells) when naïve T-cells were stimulated and treated with IgG1 and DcR3.Fc, respectively. The increase between FasL + IgG1 and FasL + DcR3.Fc groups is also obvious. Thus, DcR3.Fc clearly upregulated the dose-dependent FasR expression. This event was observed with DcR3 at 5 μg/ml, and it peaked at 10 μg/ml (**Figure 4H**).

FasL and TRAIL are essential for IFN-γ and TNF-α production in CD4^+^ Teffs to promote Th1 polarization to clean alloantigens. Interestingly, DcR3 dose-dependently suppressed Th1-cytokine TNF-α secretion (**Figure 4I**), IFN-γ at the mRNA level (**Figure 4J**) as well as time-dependent IFN-γ secretion attenuation (**Figure 4K**).

### DcR3 Might Induce Teff Apoptosis Through FasR/FADD/Caspase 8 Pathway Rather Than TNFR1/TRADD Pathway

**Figure 5:**
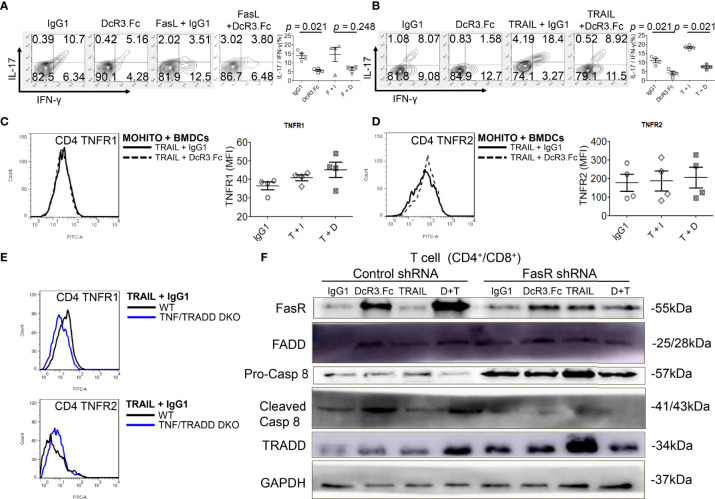
Decoy receptor 3 (DcR3) suppresses the tumor necrosis factor receptor-associated factor 1 (TNFR1) pathway and upregulates FasR/Fas-associated protein with death domain (FADD)/caspase 8 pathway. **(A, B)** Interleukin-17 and interferon-γ production by bone marrow-derived dendritic cell (BMDC)-activated MOHITO T-cells co-treated with or without FasL/TRAIL was measured using intracellular cytokine staining. Right panel, a dot graph of T-cells treated with IgG1, DcR3.Fc, FasL/TRAIL+IgG1, and FasL/TRAIL+DcR3.Fc for 36 h. **(C)** TNFR1 expression. Right panel, mean fluorescence intensities (MFI) of isotype controls and stimulated samples. **(D)** TNFR2 expression. Right panel, MFI of isotype controls and stimulated samples. **(E)** TNFR1 and TNFR2 expression in CD4^+^ T-cells of WT and TNF/TNFR1-associated death domain protein (TRADD) double knockout (DKO) mice. The cells were double-stained with anti-mCD4-allophycocyanin–cyanine7 (cy7)/TNFR1-FITC or TNFR2-FITC. **(F)** FasR/FADD/TRADD/caspase 8 pathway and Fas shRNA on the MOHITO cell lines. All experimental models among female WT, female TNF/TRADD DKO, and MOHITO (immortalized CD4^+^CD8^+^ T-cell line, BALB/c origin) T-cells are BMDCs (male C57BL/6 origin) + LPS-activated method for 36 h. Lower pro-caspase 8 was found in immortalized T-cells co-treated with IgG1, DcR3.Fc, TRAIL+IgG1, TRAIL+DcR3.Fc, and FasR shRNA; immortalized T-cells co-treated with IgG1, TRAIL, and control shRNA indicate no triggering apoptosis of effector T (Teff) cells. F, FasL; I, human IgG1; D, DcR3.Fc; T, TRAIL; DcR3.Fc 10 μg/ml; FasL 100 ng/ml; TRAIL 100 ng/ml.

DcR3 decreased TNFR1 downstream cytokines IL-17 and IFN-γ in the MOHITO cells based on intracellular cytokine staining (**Figures 5A, B**). TNFR1 expression was unaffected after DcR3.Fc treatment (**Figure 5C**). However, unexpectedly, DcR3.Fc-treated and BMDC-activated MOHITO cells predominantly expressed TNFR2 (mean difference of mean fluorescence intensities of TNFR2 and TNFR1 under TRAIL + IgG1 and TRAIL + DcR3.Fc treatment: TNFR2 16.65 *vs.* TNFR1 4.4, **Figures 5C, D**). TNFR2 expression in the CD4^+^ MOHITO cells after DcR3.Fc treatment appeared to be distributed with TNF/TRADD DKO than that in the WT murine CD4^+^ T-cells (**Figure 5E**).

Furthermore, death-inducing signaling complex (DISC) formation *in vitro* by immunoblotting the MOHITO cells was used to detect the following: FasR, Fas-associated protein with death domain (FADD), pro-caspase 8, active caspase-8, and TRADD in protein complex (**Figure 5F** and **Supplementary Figure S12**). Co-treatment of MOHITO with control shRNA and DcR3.Fc at 10 μg/ml showed enhanced signals of autoradiograph regarding FasR, FADD, and cleaved caspase-8. In contrast, upregulation of FasR recruiting FADD/cleaved caspase-8 by DcR3.Fc was abrogated after co-treating with Fas shRNA (**Figure 5F** and **Supplementary Figure S12**). Conversely, the TRADD protein expression in the T-cells co-treated with DcR3.Fc and control shRNA was weaker than those in T-cells co-treated with DcR3.Fc and FasR shRNA (**Figure 5F** and **Supplementary Figure S12**).

### Both High DcR3 Expression and Absence of TNF/TRADD Signaling Pathway Seem to Influence TNFR2 Phenotype *In Vivo*

**Figure 6:**
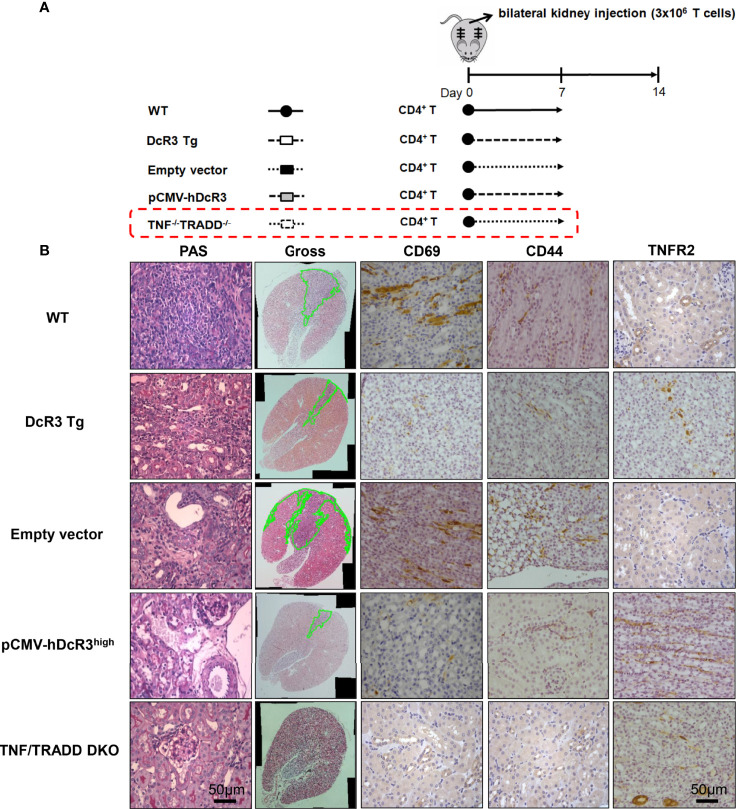
Transgenic decoy receptor 3 (DcR3 Tg), tumor necrosis factor (TNF)/TNF receptor-associated factor 1-associated death domain protein (TRADD) double knockout (DKO), wild-type (WT) mice with empty vector or human DcR3 plasmid injection were tested to verify the presence or absence of TNF/TRADD signaling pathway during TNFR2 phenotype. **(A)** The mice were divided into five groups including WT, DcR3 Tg, and WT mice with hydrodynamics-based gene delivery through the tail vein with empty vector or pCMV-hDcR3. All five groups concurrently received 3 × 10^6^ activated T-cell injections in the bilateral kidney cortex. **(B)** Periodic acid–Schiff, CD69, CD44, and TNFR2 immunohistochemical staining of WT, DcR3 Tg, and TNF/TRADD DKO mice and mice with empty vector or hDcR3 plasmid injection.

To determine whether DcR3 promotes TNFR2 expression *in vivo*, we used the other ACR TNF/TRADD DKO mice (**Figure 6A**). The CD69 and CD44 levels were low, but TNFR2 expression was high in infiltrating T-cells of both DcR3 Tg and TND/TRADD DKO mice (**Figure 6B**).

### High DcR3 Gene Expression in Human Kidney Allograft Accurately Predicts ACR

**Figure 7:**
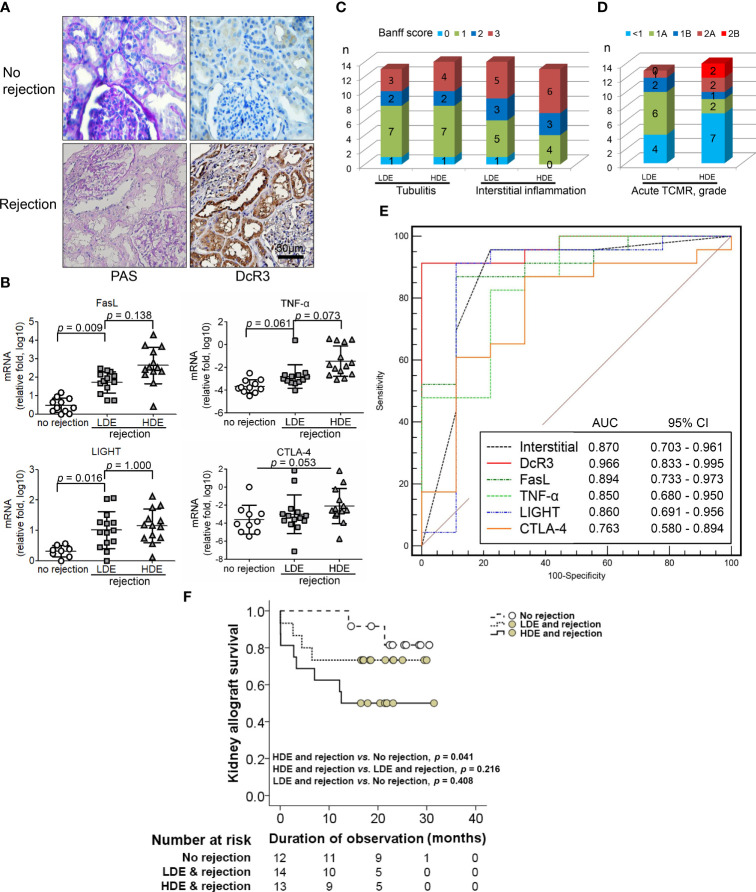
Correlation of decoy receptor 3 (DcR3) molecule with acute T-cell-mediated rejection. **(A)** Periodic acid–Schiff and DcR3 immunohistochemical staining of recipients without and with rejection. **(B)** Apoptosis, inflammatory, Teff, and Treg gene expression were correlated with no rejection, low DcR3 expression (LDE), and high DcR3 expression (HDE). **(C)** HDE was associated with Banff tubulitis, interstitial inflammation, and **(D)** TCMR grading severity (*n* = 27). **(E)** Predictive value of DcR3 gene expression in recipients with acute TCMR after comparison with pathological findings and TNF ligand and receptor-related gene expression in humans with biopsy-proven acute cellular rejection. **(F)** Survival curves in renal transplant recipients with no rejection and rejection with high and low DcR3 expression. Kaplan–Meier cumulative curves for composite endpoints: serum creatinine doubling or graft failure. HDE, high DcR3 expression; LDE, low DcR3 expression; HDE and LDE were determined by the median qPCR values ​​of human kidney transplant biopsy specimens.

Human RTRs without rejection (*n* = 12) and with biopsy-proven ACR (BPACR, *n* = 27) (**Figure 7A**) were recruited in this experiment. DcR3 expression was high in patients with BPACR. Moreover, patients with BPACR had significantly high DcR3, apoptotic gene, inflammatory gene, co-stimulatory, and co-inhibition gene expressions (**Supplementary Table S4**) in their kidney allografts. Tissue HDE was significantly correlated with Fas, FasL, TNF-α, IL-2, IL-12a, IL-4, and LIGHT (**Figure 7B** and **Supplementary Figure S13**). Tubulitis, interstitial inflammation (**Figure 7C**), and grading of acute TCMR were also severe (**Figure 7D**). Furthermore, DcR3 had the best predictive value (area under the curve = 0.966; 95% confidence interval = 0.833–0.995) for acute TCMR after comparing the pathologic findings, TNF ligands, and receptor-related gene expressions in the patients with BPACR (**Figure 7E**). After performing a prospective study, it is clear that patients with HDE and allograft rejection had the poorest graft survival (**Figure 7F**).

## Discussion

This is the first study to demonstrate that DcR3 upregulation during ACR induces negative feedback to suppress inflammation. Our findings are in line with the finding that TNF family members generate bidirectional signals. Both FasL and TRAIL co-stimulate Ag-specific CD4^+^ and CD8^+^ T-cell proliferation in B6.WT (27, 31–33); however, DcR3 induces T-cell co-inhibition (12). In our established *in vitro* model of CD4^+^/CD8^+^ T-cells with Y-chromosome and allogeneic stimulation, a high DcR3 dose (10 μg/ml) suppressed T-cell priming through FasL/TRAIL-mediated reverse signaling (12, 34) after observing limited effects of DcR3 on RTECs co-cultured with TCR/IL-2-triggered T-cells showing generalized partial apoptosis resistance.

**Figure 8:**
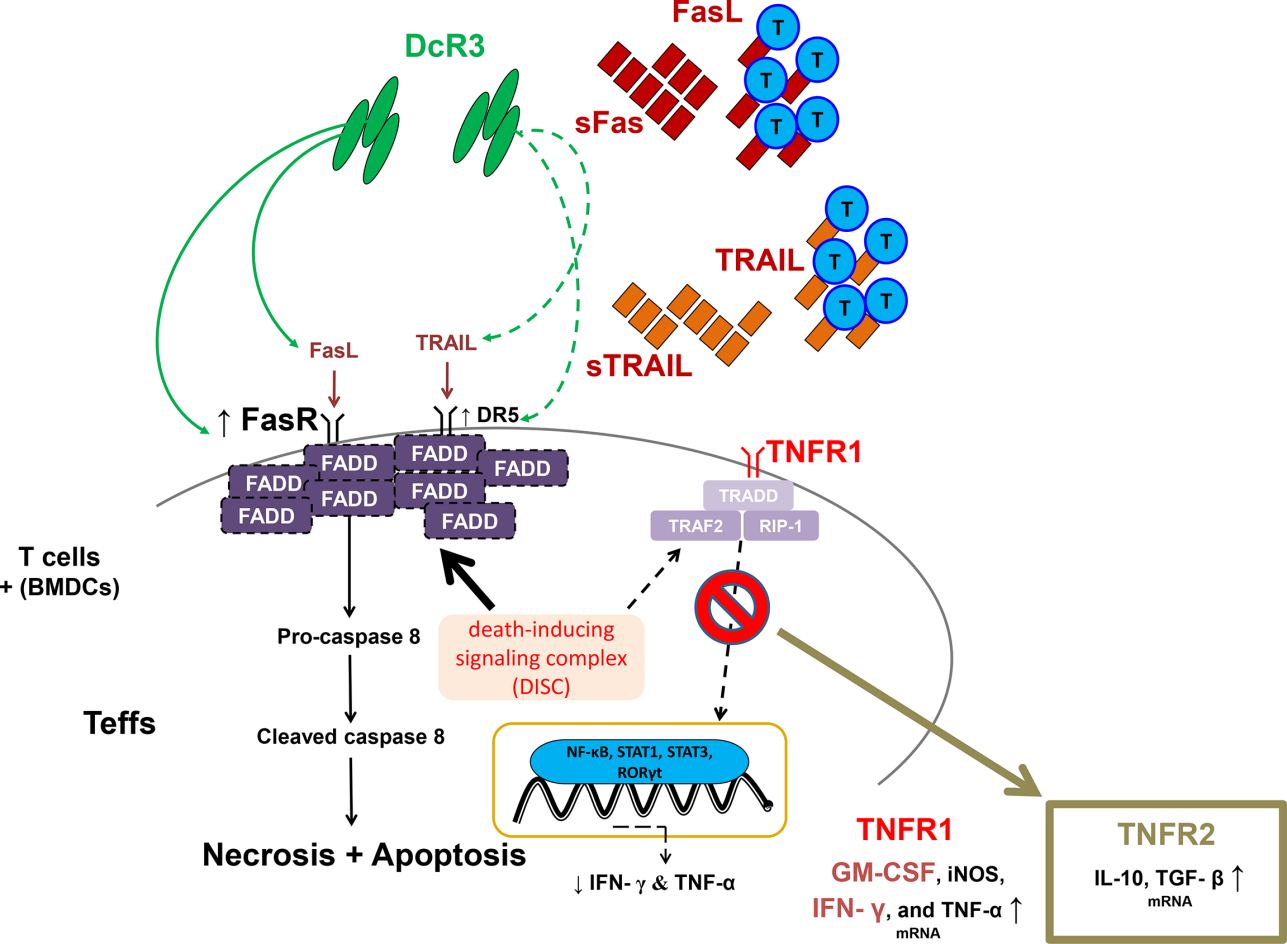
A simple schematic diagram illustrating the potential effects of decoy receptor 3 on T-cells in the acute T-cell-mediated rejection. FADD, Fas-associated protein with death domain; GM-CSF, granulocyte–macrophage colony-stimulating factor; iNOS, inducible nitric oxide synthase; RIP1, receptor-interacting serine/threonine kinase 1; RORγt, retineic-acid-receptor-related orphan nuclear receptor gamma; STAT1, signal transducer and activator of transcription 1; STAT3, signal transducer and activator of transcription 3; TGF-β, transforming growth factor beta; TRADD, TNFR1-associated death domain protein; TRAF2, TNF receptor-associated factor 2.

Moreover, DcR3 critically suppressed Teff from differentiating to CD4^+^CD69^hi^, CD4^+^CD44^hi^, CD8^+^CD69^hi^, and CD8^+^CD44^hi^ T-cells *via* interfering with TNF ligands, resulting in a positive signal loss and activation/proliferative capacity attenuation. The anchoring effect of the Fc portion on Fc receptor-bearing cells is unlikely to trigger reverse signaling. In the cell lysate qPCR test (activated primary T-cells), DcR3 promoted Teff apoptosis through FasR/FADD/caspase 8 upregulation prone to anti-Th1 phenotype (**Figure 8**). DcR3 likely critically regulated the intragraft ratio between Teffs and Tregs, and DcR3 treatment, based on the Th2-like phenotype behaviors, could be a promising therapeutic strategy (**Supplementary Figure S14**). In our animal model, DcR3 also modulated the corresponding ligands, enhanced the Th2 immune response, and suppressed the Th1 response.

A previous study on a yeast two-hybrid cloning system (HeLa cells and mouse fetal liver stromal cells) has suggested that the C-terminal death domain of FADD (80–205) inhibits TNF-induced apoptosis without inhibiting TNF-mediated NF-κB activation (35). Moreover, DcR3.Fc might upregulate FasR and recruit FADD-mediated necrosis and apoptosis but abrogate TNFR1 downstream activation due to TRADD deficiency (36). The expression levels of death receptors, TRAIL-R2 (DR5), was also high in DcR3.Fc-treated MOHITO cells and MHC-mismatched stimulator primary T-cells (**Supplementary Figure S15**), but TNFR1 played no role in T-cell death after the DcR3.Fc treatment (**Supplementary Figure S11F**).

The TNFR2 expression pattern in the DcR3.Fc-treated CD4^+^ MOHITO cells appeared to follow TNF/TRADD DKO distribution after comparing it with WT murine T-cells. Interestingly, the kidney histology of ACR WT mice concurrently injected with pCMV-hDcR3 *via* the tail vein showed less destruction than that of TNF/TRADD DKO mice. However, the absence of TNF/TRADD signaling pathway in recipients with the alternative TNFR2 phenotype limits alloreactivity *in vivo* (37, 38). Furthermore, ACR DcR3 Tg mice represent Treg- and Th2-like transformations (**Supplementary Figure S16**). The implication of TNFR2 expression, Treg, or Th2-like transformation of T-cells seemed to have a protective role in graft rejection.

Our study has some limitations to be addressed. First, although DcR3 is beneficial for the established ACR models both *in vitro* and *in vivo*, it may be better to translate DcR3 to be a prognostic indicator or biomarker rather than a novel therapeutic tool in clinical practice. DcR3 does not exist in mouse or rat genomes. Therefore, studying its physiological functions in man-made ACR models is difficult. Second, the serum DcR3 levels in Tg or WT mice with pCMV-hDcR3 injections are extremely high to be an artificial protein. Therefore, further investigations on DcR1, DcR2, DcR3v1, or DcR3v2 expressions both in mice or rats are needed to determine if their expressions can serve as indicators for decoy function on death or TNF ligands (39). In addition, cancer occurrence possibility should be considered and observed in the DcR3 Tg or WT mice with pCMV-hDcR3 treatment because DcR3 protein was produced mainly from cancer cells as reported in previous studies. Third, many phenotypic markers, such as CD62L and CD127, did not specifically show effector or memory T-cells. Fourth, the status of cellular FADD-like IL-1β-converting enzyme-inhibitory protein was not examined on western blot, as this protein is a major regulator of DISC and FasR signaling. Although the DcR3 expression levels on western blot data were not provided, DcR3 mRNA in kidney lysates in DcR3 Tg mice was also shown to be high after comparing with WT mice under different levels of T-cell injection.

Based on the results of previous studies and the animal studies of this work, high DcR3 levels, at least the soluble form of this molecule, are immunosuppressive and protective against graft rejection. However, high DcR3 levels in the patients are associated with increased rejection risks. The possible reason for this discrepancy is that endogenous DcR3 is not high enough to cope with T-cell response modulation in human kidney allograft. In summary, *in vitro* experiments showed that high DcR3 doses protect RTECs from acute T-cell attack during T-cell priming *via* interfering with TNF ligand-mediated reverse signaling and possibly promoting Teff apoptosis through FasR upregulation prone to anti-Th1 phenotype. In the ACR animal model, DcR3 participated in T-cell immunology by suppressing the related ligands.

## Data Availability Statement

The original contributions presented in the study are included in the article/**Supplementary Material**. Further inquiries can be directed to the corresponding authors.

## Ethics Statement

The studies involving human participants were reviewed and approved by Institutional Review Board of Taichung Veterans General Hospital (No. C10187, C10187-1, C10187-2, CF11126, CF11126-1, CF11126-2, CF11126-3). The patients/participants provided their written informed consent to participate in this study. The animal study was reviewed and approved by Institutional Animal Care and Use Committee (IACUC) of the National Yang-Ming University (IACUC1001106) and Taichung Veterans General Hospital (La-1011004, La-1031225, La-1061491, La-1071577), Taiwan.

## Author Contributions

S-CW, N-JC, and D-CT conceived and designed the experiments. S-CW, M-CW, N-JC, S-LH, and D-CT performed the experiments. S-LH provided the DcR3 Tg mice. S-CW, M-CW, N-JC, and D-CT analyzed the data. S-CW, N-JC, and D-CT wrote the paper. All authors contributed to the article and approved the submitted version.

## Funding

We are deeply indebted to Taichung Veterans General Hospital, Taichung, for providing the grants for this study (TCVGH-YM1040103, TCVGH-YM1050101, TCVGH-YM1060103, TCVGH-1078201B, TCVGH-YM1070101, TCVGH-1088201B, TCVGH-YM1080103, TCVGH-1098201B, TCVGH-YM1090105, TCVGH-1108201B, and TCVGH-1118202C). This study was also supported by the Taiwan Ministry of Science and Technology (MOST 106-2314-B-075A-003 and MOST 109-2314-B-075-097-MY3) and Foundation for Poison Control (2022-04).

## Conflict of Interest

The authors declare that the research was conducted in the absence of any commercial or financial relationships that could be construed as a potential conflict of interest.

## Publisher’s Note

All claims expressed in this article are solely those of the authors and do not necessarily represent those of their affiliated organizations, or those of the publisher, the editors and the reviewers. Any product that may be evaluated in this article, or claim that may be made by its manufacturer, is not guaranteed or endorsed by the publisher.
