# Supplementary material for: Decoy Receptor 3 Suppresses T-Cell Priming and Promotes Apoptosis of Effector T-Cells in Acute Cell-Mediated Rejection: The Role of Reverse Signaling
Source: Front Immunol. 2022 Jun 2;13:879648. doi: 10.3389/fimmu.2022.879648 (PMC9201909; doi:10.3389/fimmu.2022.879648)
Supplement: Supplementary file 1 [file DataSheet_1.pdf]

SUPPLEMENTARY MATERIAL

Decoy Receptor 3 Suppresses T-Cell Priming and Promotes Apoptosis of Effector T-Cells in Acute Cell-Mediated Rejection: The Role of Reverse Signaling

Supplementary Table 1. Antibodies for the basic study.

Antibody	Source	Catalogue Number	Dilution			
**Primary**			**WB**	**IF**	**IHC**	**Flow**
Activated caspase 3	abcam	ab4051			1:500	
LIGHT	abcam				1:100	
CTLA-4	abcam				1:100	
CD44	eBioscience				1:200	
CD69	eBioscience				1:200	
TNFR2	eBioscience				1:200	
FasL	St John's Laboratory		1:500			
LIGHT	Thermo Fisher Scientific		1:1000			
TRAIL	R&D sysyems a biotechne brand	MAB1121	1:1000			
TL1A	Sigma-Aldrich		1:1000			
CD45	Sigma-Aldrich			1:400		
CD3	Sigma-Aldrich			1:400		
CD4	Sigma-Aldrich			1:400		
CD8	Bioss antibodies	bs-0648R		1:400		
CD20	LifeSpan BioSciences			1:400		
CD68	Bioss antibodies	bs-0649R		1:400		
DcR3	R&D Systems, Inc., Minneapolis, USA			1:400		
CD4-PE	eBioscience					1:200
CD4- APC/Cy7	BioLegend					1:200
CD8-APC	Elabscience					1:200
CD8- APC/Cy7	BioLegend					1:200
CD25-FITC	BioLegend					1:200
Foxp3-Alexa Fluor	BioLegend					1:200

CD44-APC	BioLegend			1:200
CD69-APC	BioLegend			1:200
FasL-intracellular	BD Pharmingen			1:200
Fas (CD95)	BioLegend			1:200
TRAIL-R2 (DR5)	Abcam			1:200
IL-17	BD Biosciences			1:200
IFN-γ	BD Biosciences			1:200
TNFR1	Proteintech	21574-1-AP		1:100
TNFR2	Proteintech	19272-1-AP		1:100
FADD	eBioscience		1:1000	
TRADD	eBioscience		1:1000	
Pro-caspase 8	eBioscience		1:1000	
Cleaved caspase 8	eBioscience		1:1000	

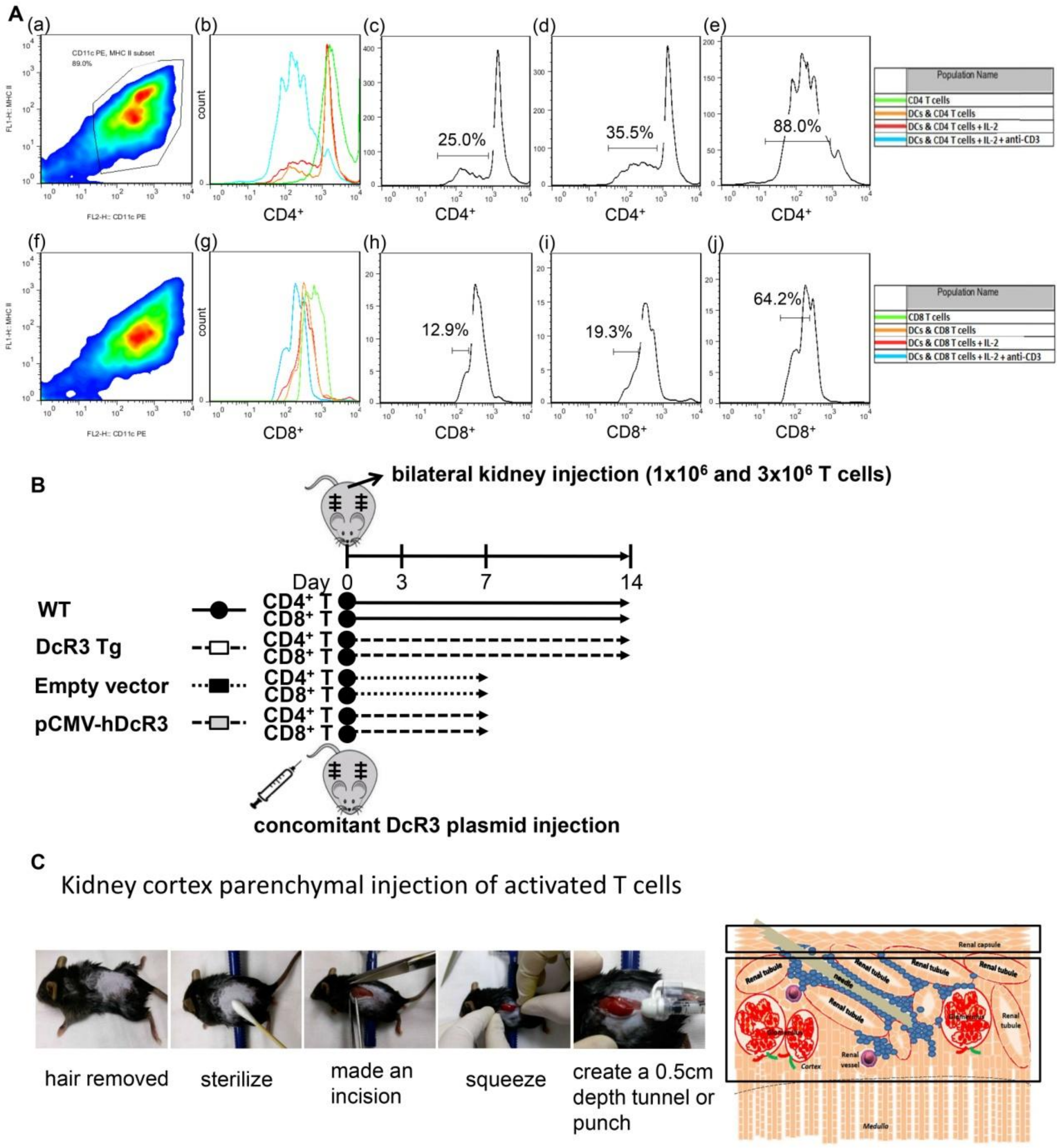

Supplementary Figure 1. (A) *In vitro* co-incubation of bone marrow-derived DCs (BMDCs) from male and $CD4^+$ or $CD8^+$ T cells from female. Activated DCs was confirmed by MHC II & CD11c-PE after 10-day stimulation (a, f); cell proliferation was confirmed with CFSE by flow cytometry on day 13 (b–e & g–j). **(B)** Mice were divided into 4 groups including wild type (WT), DcR3 Tg, and WT mice with hydrodynamics-based gene delivery through tail vein with empty vector or pCMV-hDcR3. All four groups of mice concurrently received different doses ($3x10^6$ versus $1x10^6$) of activated T-cell injection in bilateral kidney cortex. **(C)** Kidney cortex parenchymal injection of activated T cells.

Supplementary Table 2. Primer sequences for real-time polymerase chain reaction in mouse.

Target gene	5' to 3' end	Sequences	base	product
**Cytokine**				
mIL-2	F:	TGCCCAAGCAGGCCACAG	18	150bp
	R:	TTACAACAGTTACTCTGATATTG	23	
mIL-4	F:	CAGAGAGTGAGCTCGTCTG	19	158bp
	R:	GGTGCAGCTTATCGATGAATC	21	
mIL-10	F:	ATGCAGGACTTTAAGGGTTAC	21	292bp
	R:	CCTGAGGGTCTTCAGCTTC	19	
mIFN-γ	F:	CTTCCTCATGGCTGTTTCTG	20	152bp
	R:	TGTCACCATCCTTTTGCCAG	20	
miNOS	F:	CATTCTACTACTACCAGATCG	21	115bp
	R:	GCAAAGAACACCACTTTCACC	21	
mIL-12a	F:	ACATGGTGAAGACGGCCAG	19	169bp
	R:	GAAGTCTCTCTAGTAGCCAG	20	
**TNF ligands**				
mFasL	F:	TCCGTGAGTTCACCAACCAAA	21	120bp
	R:	GGGGGTTCCCTGTTAAATGGG	21	
mTRAIL	F:	CAACTCCGTCAGCTCGTTAGAAAG	24	200bp
	R:	CGGCCCAGAGCCTTTTCATTC	21	
mLIGHT	F:	GCTCTTACTGACTGGCATGAG	21	105bp
	R:	CGCAGCTCTAGGAGCATGTG	20	
**TNF receptors**				
mFasR	F:	GGGTCCATTGAGGATGTCCTT	21	139bp
	R:	TTGGGAGGAACTTTGCCTTTG	21	
mDR5	F:	GGGAAGAAGATTCTCCTGAGATGTG	25	98bp
	R:	ACATTGTCCTCAGCCCCAGGTCG	23	
GAPDH	F:	GCATCCACTGGTGCTGCC	18	146bp
	R:	TCATCATACTTGGCAGGTTTC	21	

TRAIL, TNF-related apoptosis-inducing ligand; LIGHT, homologous to lymphotoxin, exhibits inducible expression and competes with HSV glycoprotein D for binding to herpes virus entry mediator, a receptor expressed on T lymphocytes; GAPDH, glyceraldehydes 3-phosphate dehydrogenase.

Supplementary Table 3. Primer sequences for real-time polymerase chain reaction in human.

Name	Sequence		base	Tm	product
**Cytokine**					
hIL-2	Forward	TCCTGTCTTGCATTGCACTAAG	22	60.6	161bp
	Reverse	CATCCTGGTGAGTTTGGGATTC	22	60.6	
hIL-4	Forward	CGGCAACTTTGTCCACGGA	19	62.8	111bp
	Reverse	TCTGTTACGGTCAACTCGGTG	21	61.7	
hTNF-α	Forward	GTACGCGGAGTGGCAGAAA	19	62.3	206bp
	Reverse	CAGAGGACGTTGCAGTAGC	19	60.2	
DcR3	Forward	GGCTGGTGTGTTGACTGCT	19	62.5	98bp
	Reverse	GTTGGAGCCTCATGGACTGG	20	62.2	
hIL-12a	Forward	ATGGCCCTGTGCCTTAGTAGT	21	62.7	77bp
	Reverse	AGCTTTGCATTCATGGTCTTGA	22	60.5	
**Apoptosis**					
hFas	Forward	AGATTGTGTGATGAAGGACATGG	23	56.8	175bp
	Reverse	TGTTGCTGGTGAGTGTGCATT	21	60.6	
hFasL	Forward	CTCCGAGAGTCTACCAGCCA	20	62.3	121bp
	Reverse	TGGACTTGCCTGTTAAATGGG	21	60.2	
**T cell activation**					
hLIGHT	Forward	ATACAAGAGCGAAGGTCTCACG	22	61.8	102bp
	Reverse	CTGAGTCTCCCATAACAGCGG	21	61.9	
hCTLA-4	Forward	CATGATGGGGAATGAGTTGACC	22	60.7	92bp
	Reverse	TCAGTCCTTGGATAGTGAGGTTC	23	60.8	
hPD1	Forward	ACGAGGGACAATAGGAGCCA	20	62.2	160bp
	Reverse	GGCATACTCCGTCTGCTCAG	20	62.1	
GAPDH	Forward	CTGGGCTACACTGAGCACC	19	62.0	101bp
	Reverse	AAGTGGTCGTTGAGGGCAATG	21	62.9	

TNF-α, tumor necrosis factor-alpha; DcR3, decoy receptor 3; LIGHT, homologous to lymphotoxin, exhibits inducible expression and competes with HSV glycoprotein D for binding to herpes virus entry mediator, a receptor expressed on T lymphocytes; CTLA-4, cytotoxic T-lymphocyte-associated protein 4; PD-1, programmed cell death protein 1; GAPDH, glyceraldehydes 3-phosphate dehydrogenase.

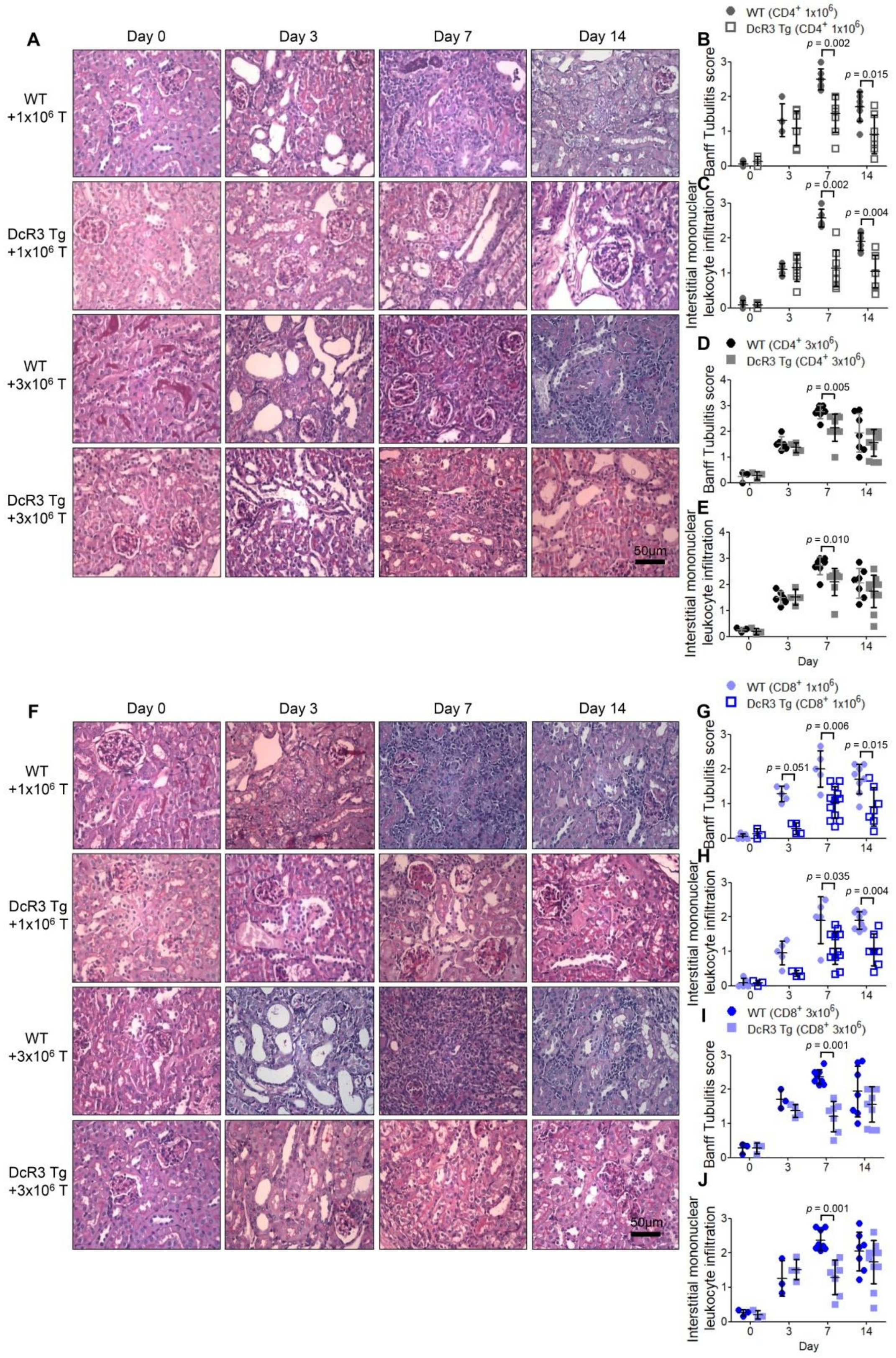

Supplementary Figure 2. Dose- and time-dependent manner of activated T cells in kidney rejection model. (A) PAS staining (400x) in WT and DcR3 Tg mice with $1x10^6$ or $3x10^6$ $CD4^+$ T-cell injection. **(B, D)** Banff t score. **(C, E)** Banff i score. **(F)** PAS staining (400x) in WT and DcR3 Tg mice with $1x10^6$ or $3x10^6$ $CD8^+$ T-cell

injection. **(G, I)** Banff t score. **(H, J)** Banff i score.

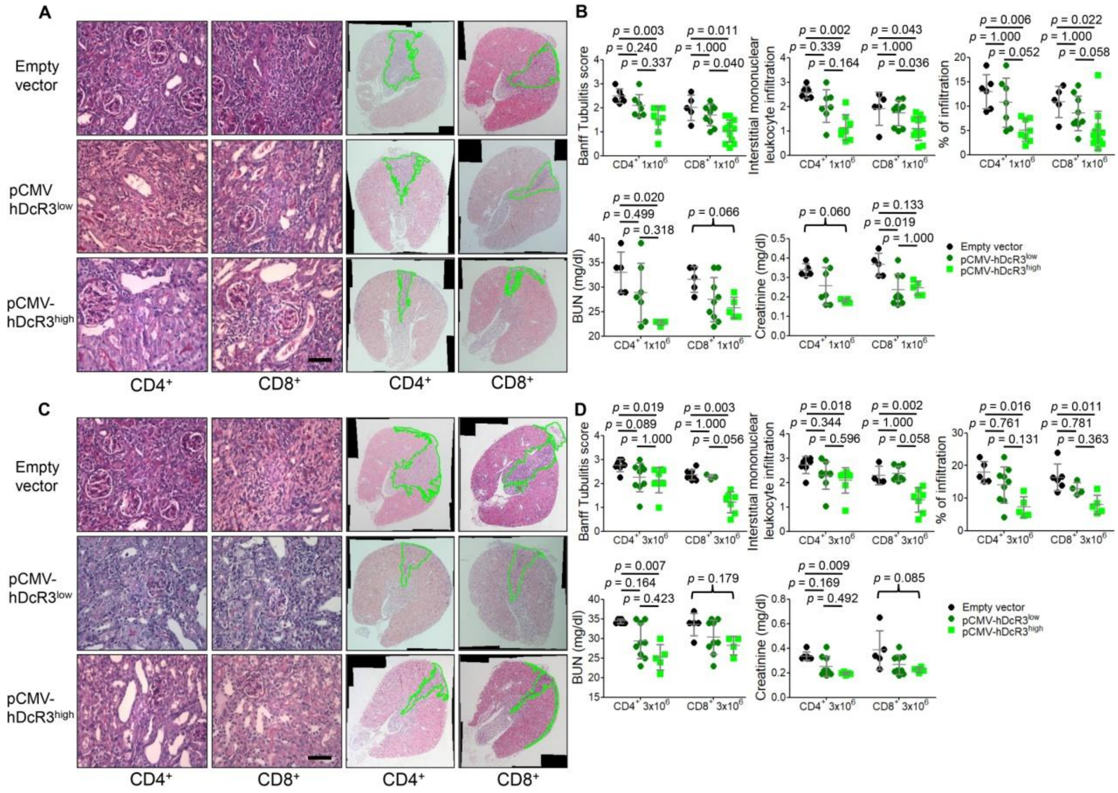

Supplementary Figure 3. Therapeutic effects of DcR3 on low and high doses of activated T cells ($CD4^+$ and $CD8^+$) in kidney rejection model. **(A)** PAS staining (400x) and **(B)** Banff t, i score, and percentage of infiltration according to histopathologic findings and renal function in WT mice treated with human DcR3 plasmid and concurrently with $1x10^6$ T-cell injection of bilateral kidney cortex. **(C)** PAS staining (400x) and **(D)** Banff t, i score, and percentage of infiltration according to histopathologic findings and renal function in WT mice treated with human DcR3 plasmid and concurrently with $3x10^6$ T-cell injection of bilateral kidney cortex. Kidneys were harvested at day 7 post injection (at least 5 mice per group). The Kruskal–Wallis test, followed by Bonferroni *post-hoc* analysis was used for multiple testing **(B, D)**.

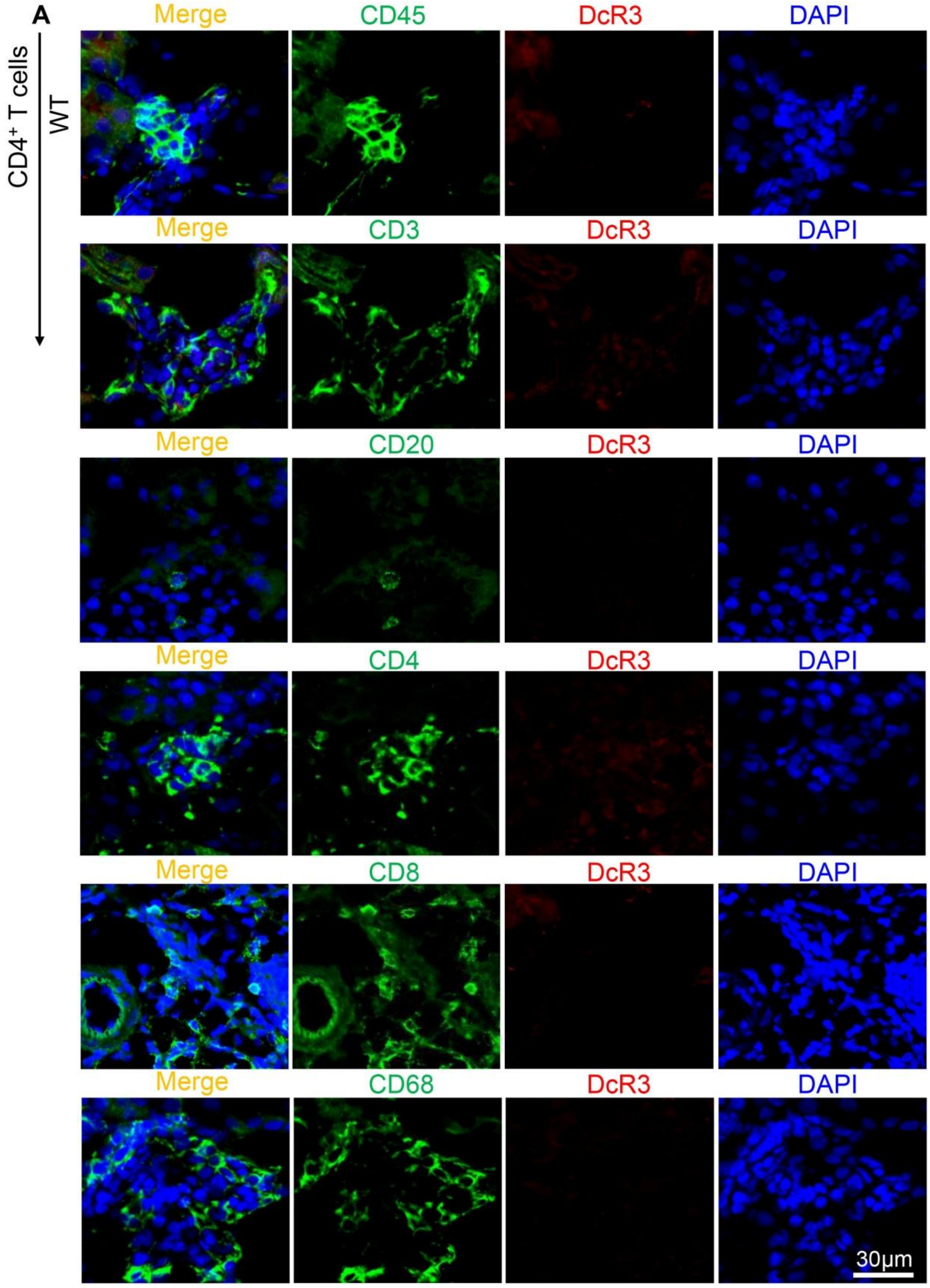

Supplementary Figure 4. DcR3 involved in T-cell immunobiology. (A) WT mice with activated $CD4^+$ T-cell injection.

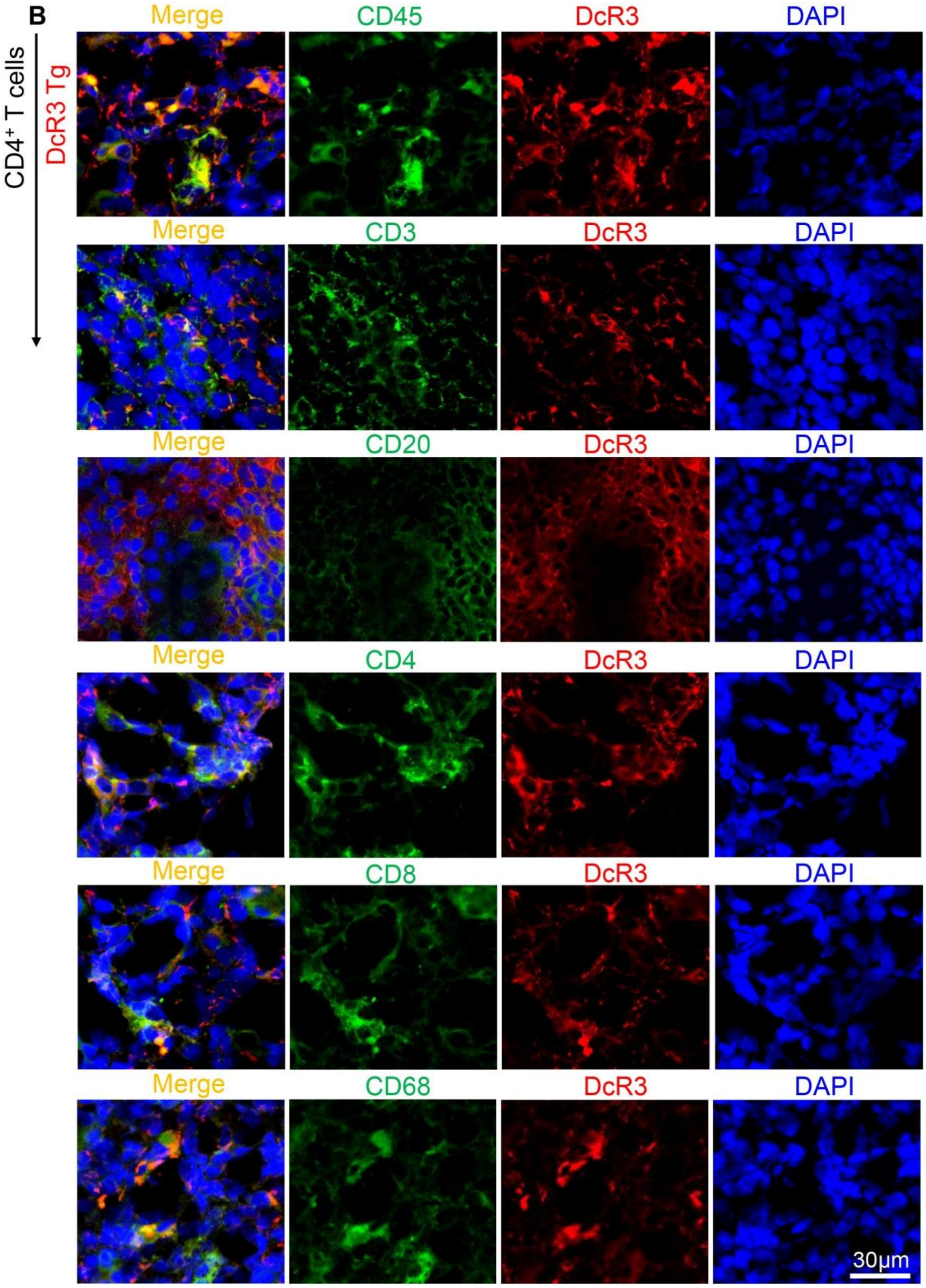

Supplementary Figure 4 (cont. 1). DcR3 involved in T-cell immunobiology. (B) DcR3 Tg mice with activated $CD4^+$ T-cell injection.

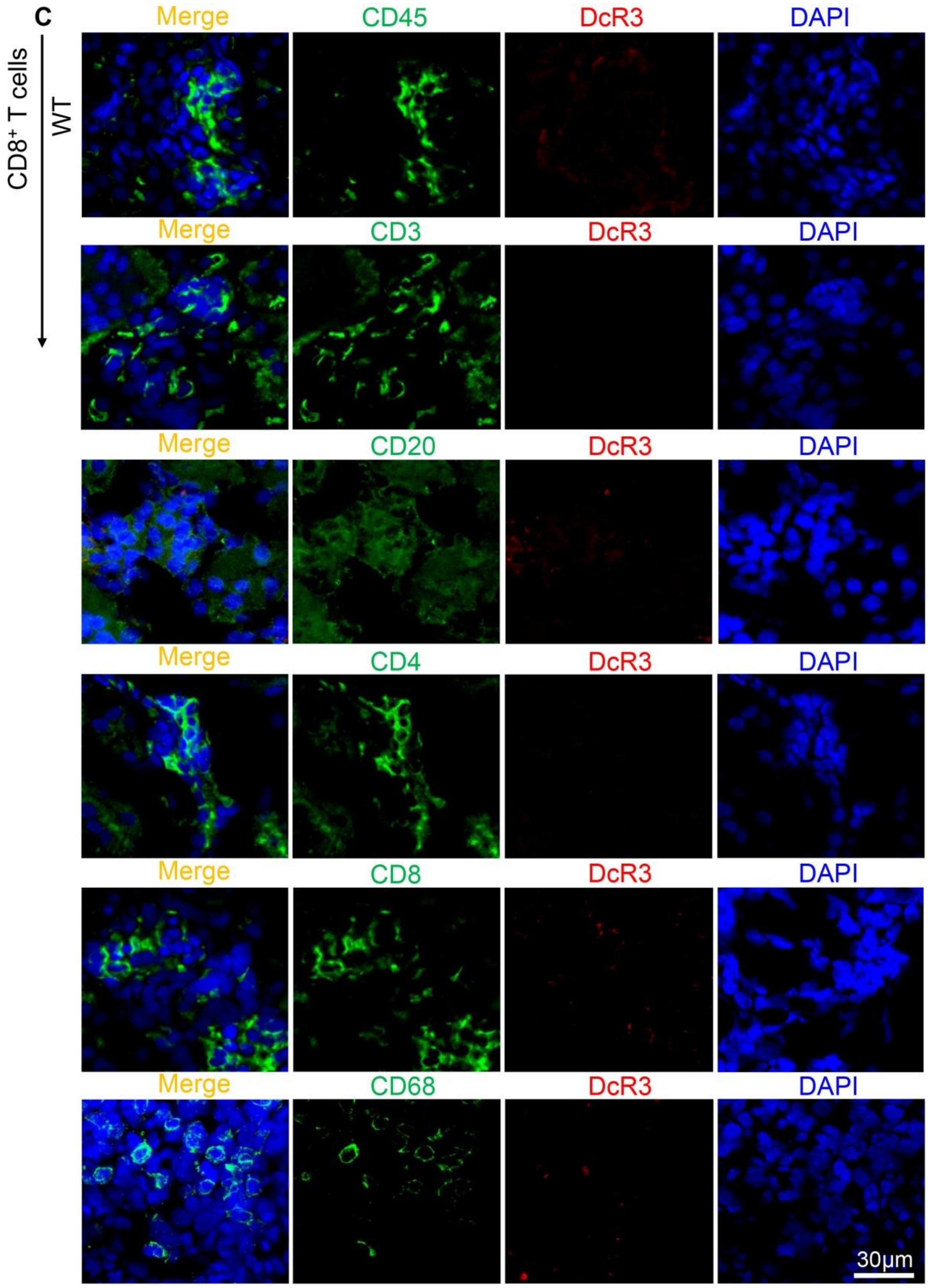

Supplementary Figure 4 (cont. 2). DcR3 involved in T-cell immunobiology. (C) WT mice with activated $CD8^+$ T-cell injection.

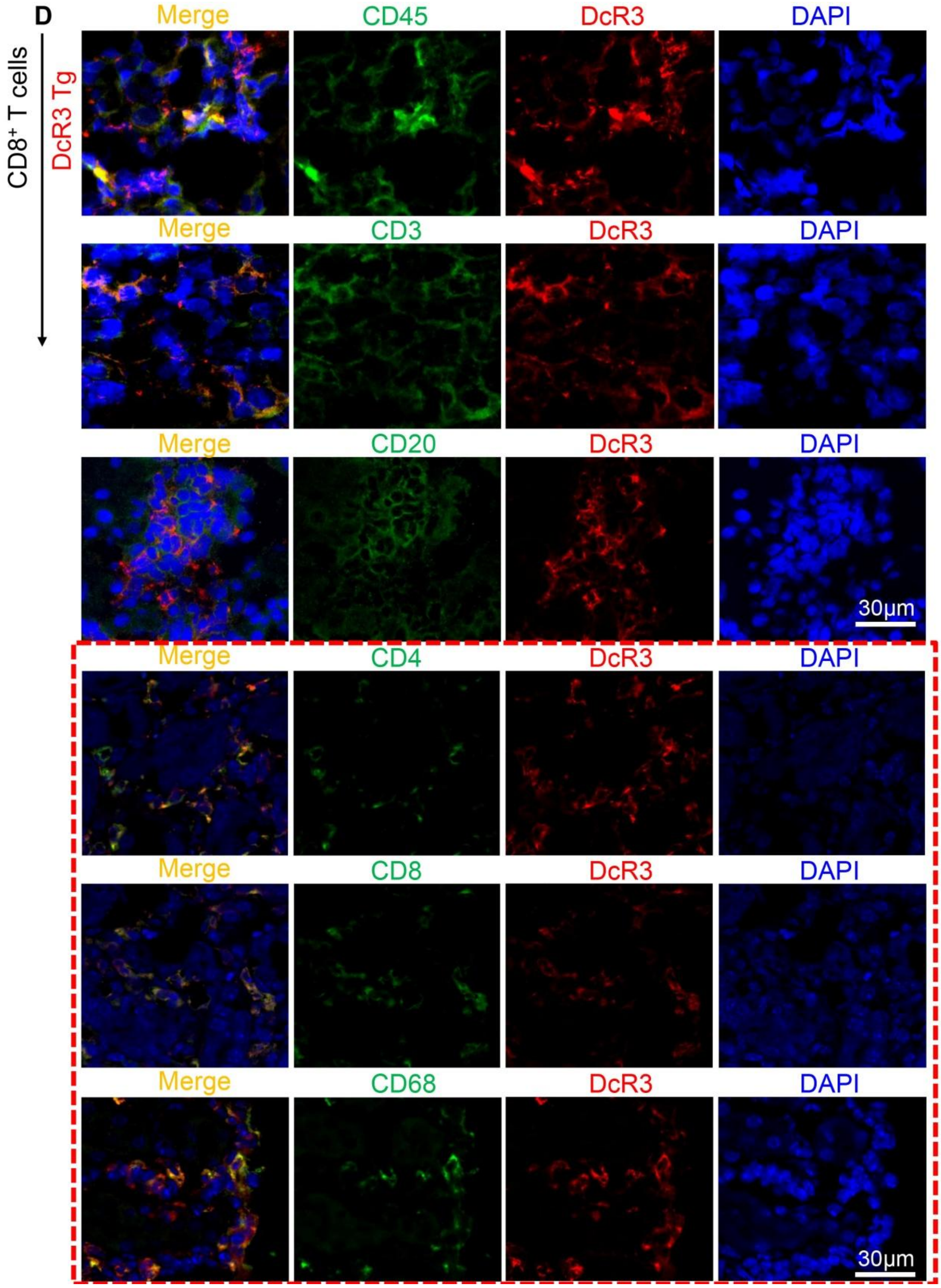

Supplementary Figure 4 (cont. 3). DcR3 involved in T-cell immunobiology. (D) DcR3 Tg mice with activated $CD8^+$ T-cell injection.

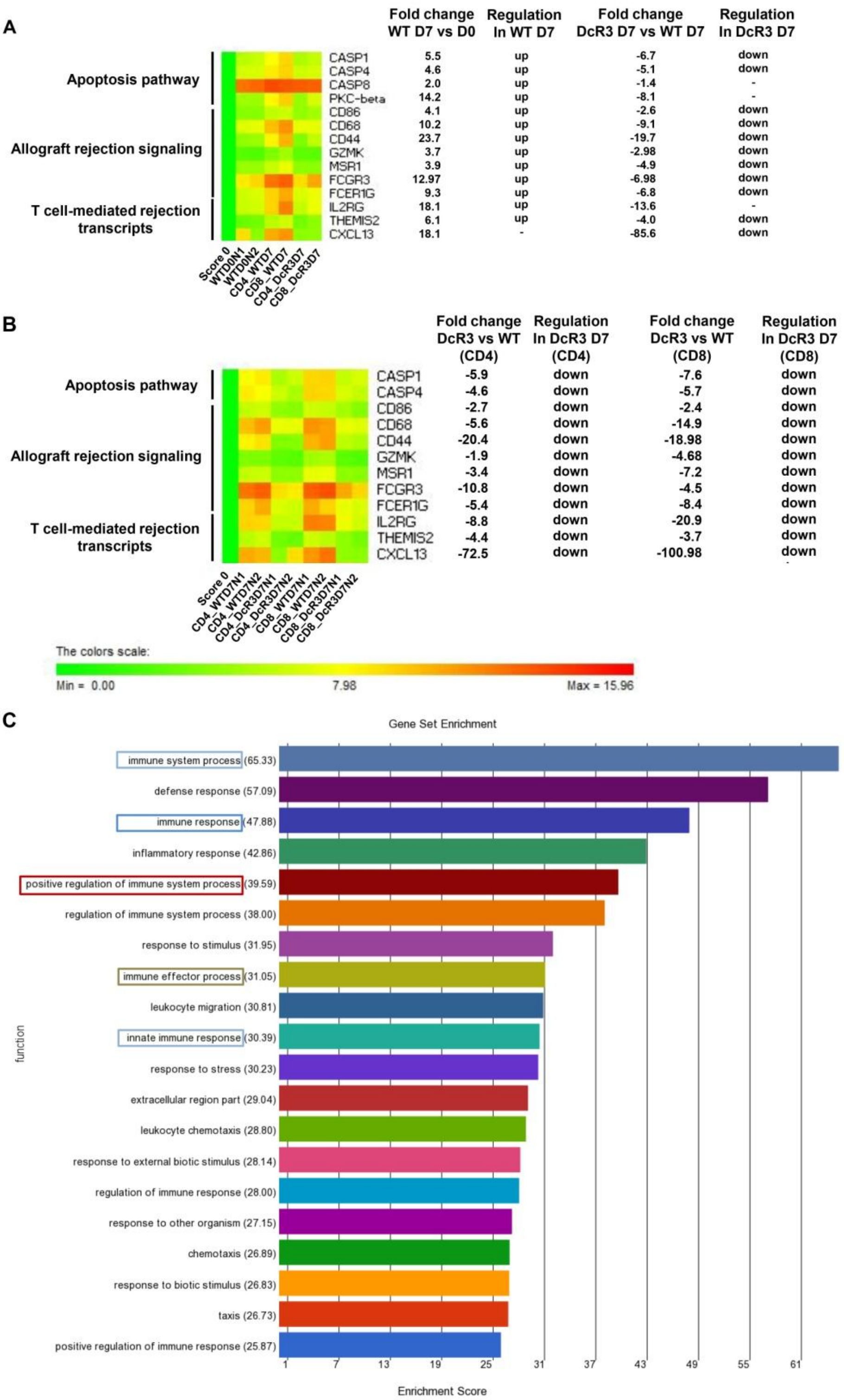

Supplementary Figure 5. mRNA microarray. Mouse kidney tissues were received RNA quality check by Agilent Bioanalyzer 2100, and then those samples were analyzed by HT07 - Clariom™ S Assay. **(A)** Heatmap and cluster gene expression between day 7 and day 0 ACR WT mice; between ACR WT and ACR DcR3 Tg mice at Day 7. **(B)** Heatmap and cluster gene expression between $CD4^+$ or $CD8^+$-related ACR WT mice at Day 7 and ACR DcR3 Tg mice at Day 7. **(C)** Top 20 Gene

Ontologies analysis between ACR WT and DcR3 Tg mice.

Results: To elucidate the potential immune regulatory mechanism and confirm the activated T-cell injection-induced kidney rejection *in vivo*, we analyzed gene expression profiles of kidney lysates from ACR WT and ACR DcR3 Tg mice by RNA sequencing and transcript microarray. We first checked the degree of overlap of differentially expressed genes among ACR WT mice at day 0, day 7, and subsequently ACR DcR3 Tg mice and ACR WT mice at day 7. As shown in **Supplemental Figure 5A**, 3 major groups (apoptosis-related genes, allograft rejection signaling, T cell-mediated rejection transcripts) of significantly differentially expressed between fold changes ≥2 or <-2. Next, heatmap and cluster gene expression showed that DcR3 turned down related gene expression and signaling pathway between ACR WT and ACR DcR3 Tg mice at Day 7. **Supplemental Figure 5B** still showed the above 3 major groups of expression between activated $CD4^+$ and $CD8^+$ T-cell injection, but there are more up-regulated or down-regulated genes, such as $CD4^+$ gene expression, $CD8^+$ cytotoxic T gene expression, leukocyte surface antigen (CD53), monocyte surface marker (CD14), myeloid lineage activating pathway, immune and inflammatory process, Toll-like receptor (TLR) family, renal tubular markers, genes for monocyte attractant and complement activation, epithelial-mesenchymal transition, TGF-β, and metabolic switch in Th17 cells (data not shown).

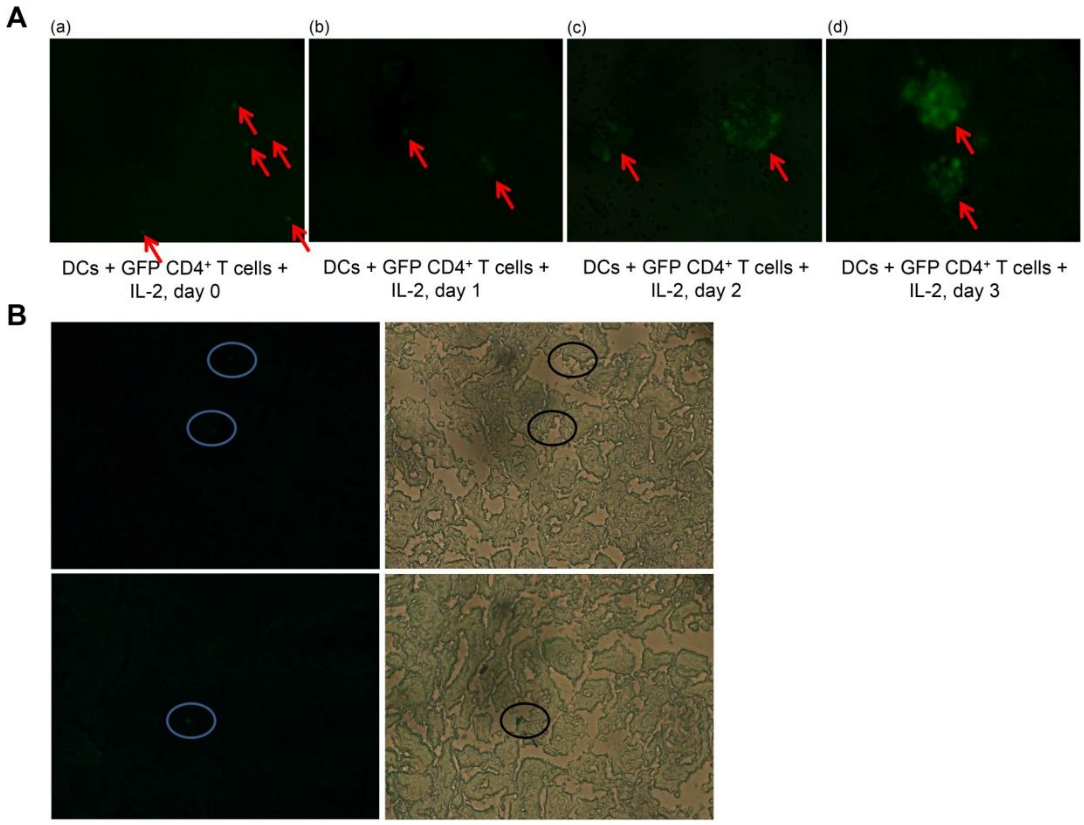

Supplementary Figure 6. Recipient-derived T cells and residual small amount of donor-derived activated T cells in day 7 of ACR mice after CD4$^+$ GFP T-cell injection. **(A)** *In vitro* co-incubation of bone marrow-derived DCs (male) and CD4$^+$ GFP T cells (female). **(B)** Frozen section and fluorescence microscope of the scarified male mouse at day 7.

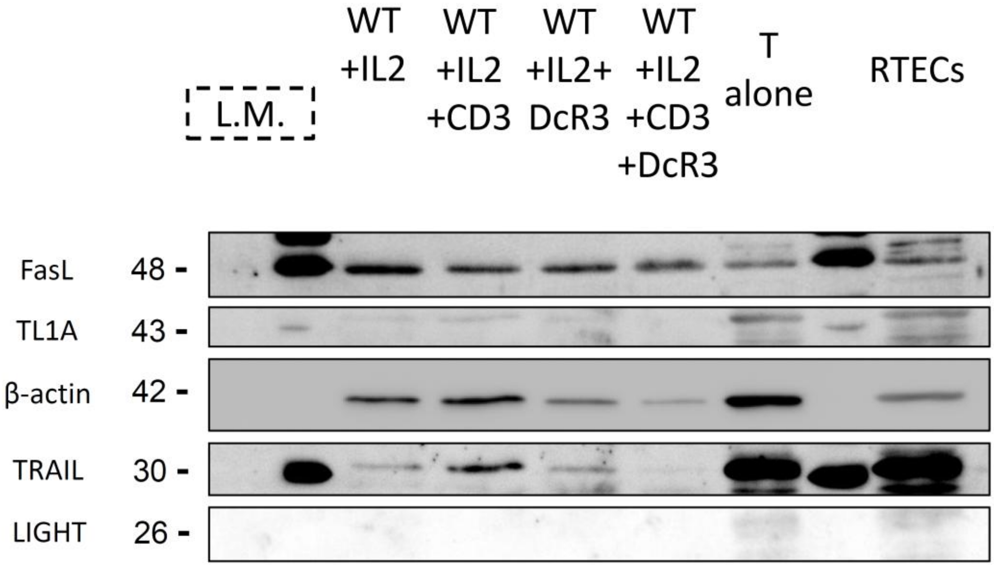

Supplementary Figure 7. FasL and TRAIL were detectable on Western blot of T-cell culture with different reagents.

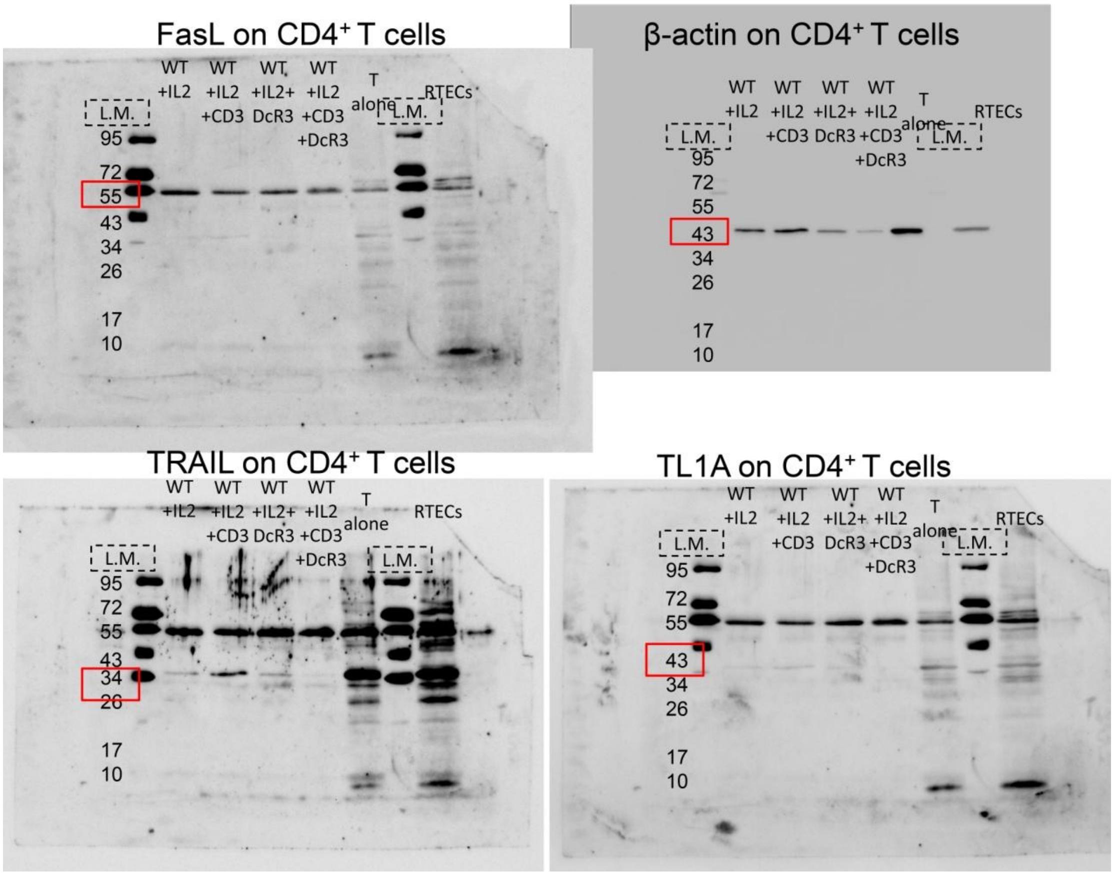

Supplementary Figure 7(A). Full gel pictures of Western blots for TNF ligands of T-cell culture with different reagents. L.M., marker.

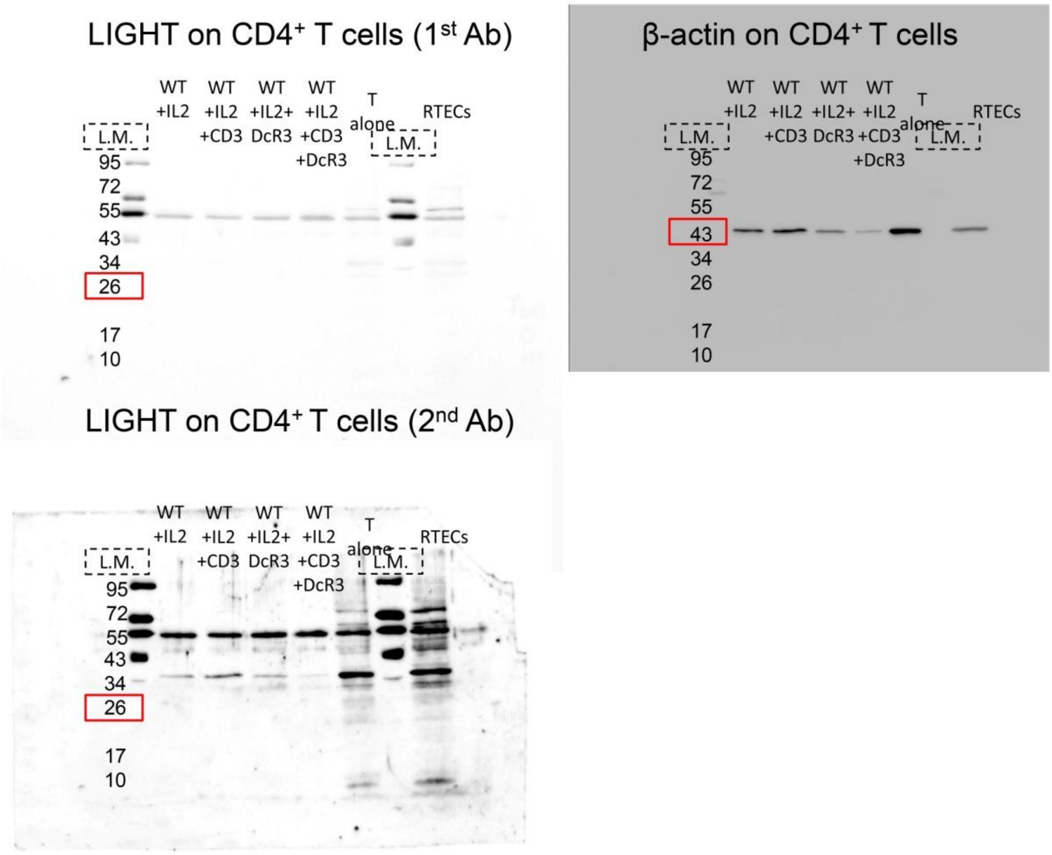

Supplementary Figure 7(B). Full gel pictures of Western blots for TNF ligands of T-cell culture with different reagents. L.M., marker.

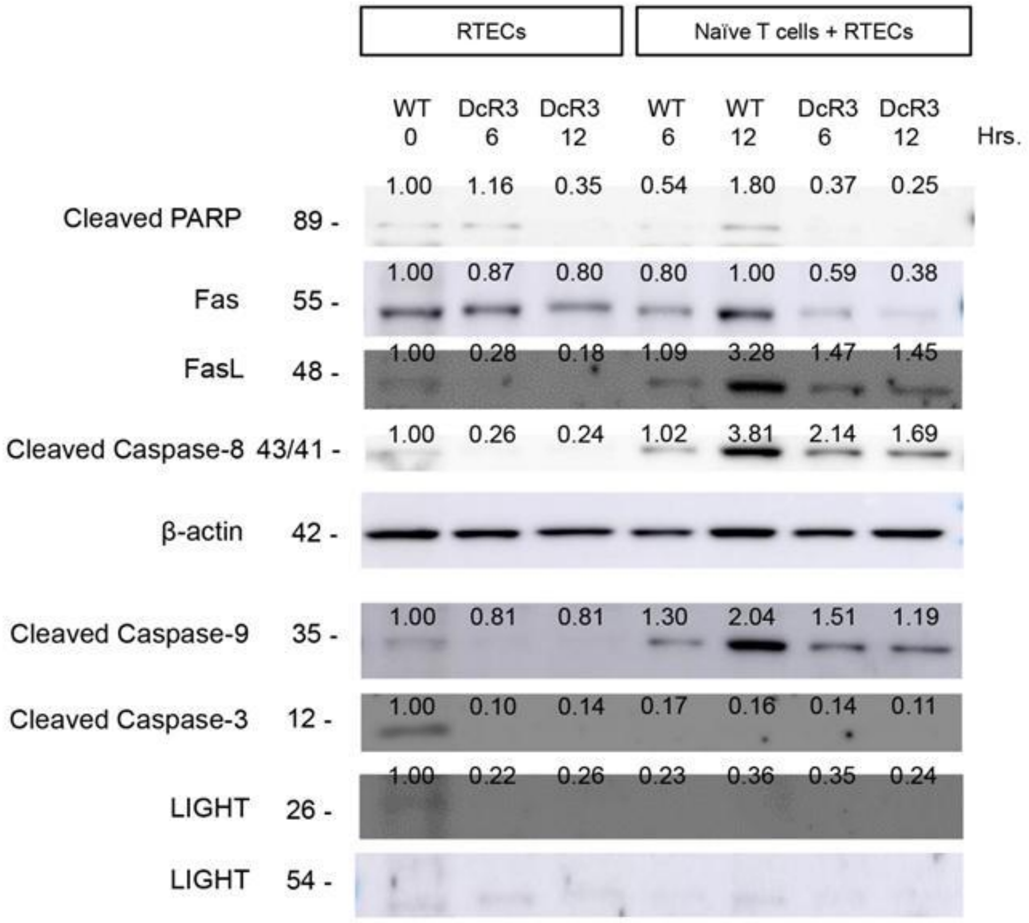

Supplementary Figure 8. Western blot showed the protective effect of DcR3 on RTECs transwell co-cultured with naïve T cells when DcR3.Fc added.

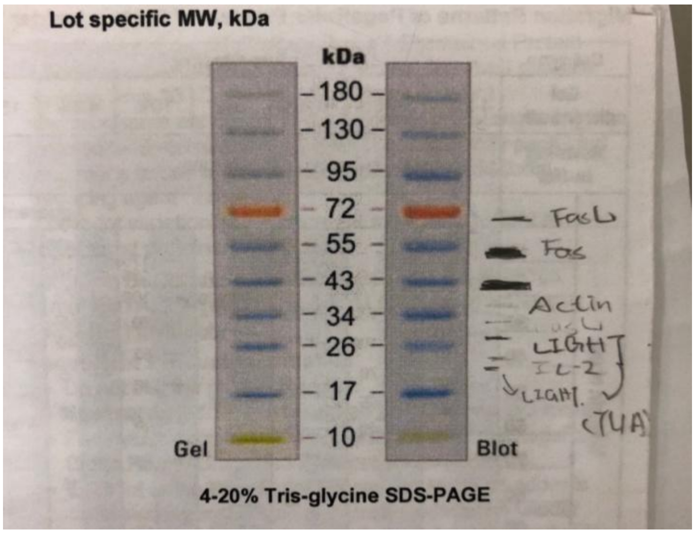

Supplementary Figure 8(A). Loading markers for apoptotic pathway of T-cell culture with different reagents. L.M., marker.

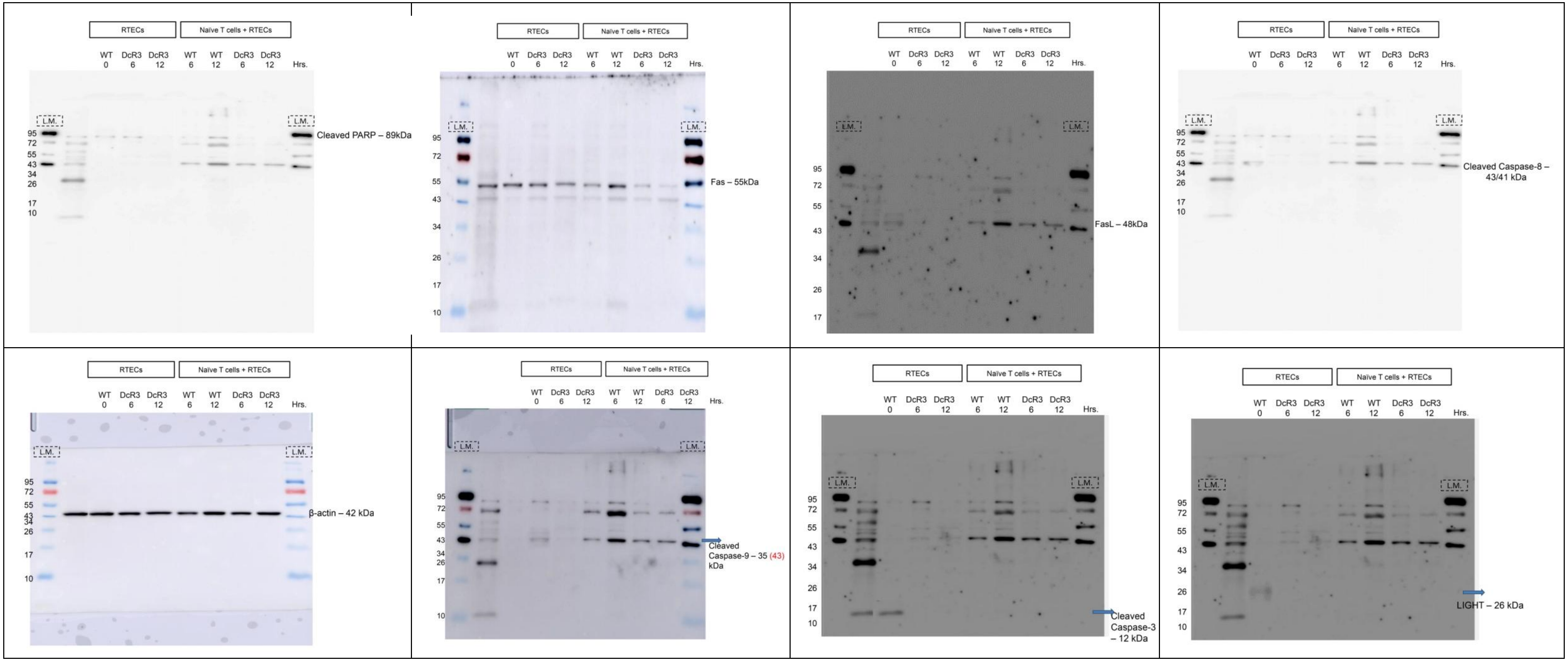
RTECs
Naïve T cells + RTECs
WT 0
DcR3 6
DcR3 12
WT 6
WT 12
DcR3 6
DcR3 12
Hrs.
L.M.
Cleaved PARP – 89kDa
Fas – 55kDa
FasL – 48kDa
Cleaved Caspase-8 – 43/41 kDa
β-actin – 42 kDa
Cleaved Caspase-9 – 35 (43) kDa
Cleaved Caspase-3 – 12 kDa
LIGHT – 26 kDa
95
72
55
43
34
26
17
10

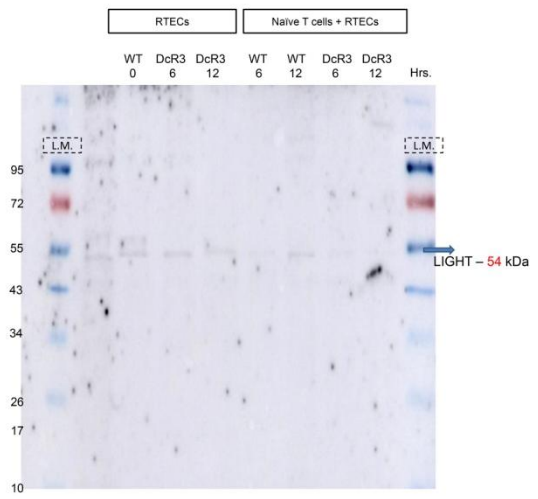

Supplementary Figure 8(B). Full gel pictures of Western blots for apoptotic pathway of T-cell culture with different reagents. L.M., marker.

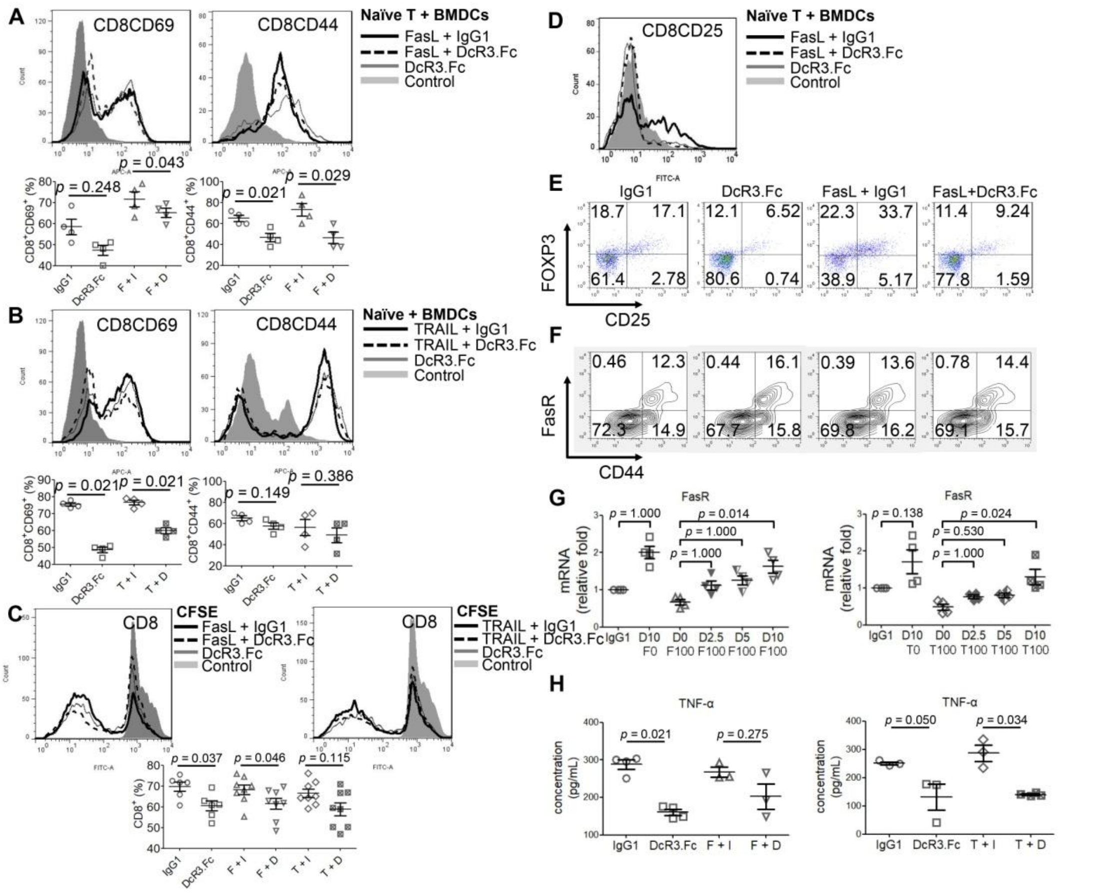

Supplementary Figure 9. Decoy receptor 3 (DcR3) suppresses FasL/TRAIL-related T-cell activation and proliferation, promotes effector T-cell (Teff) necrosis and apoptosis, and inhibits pro-inflammatory cytokines. (A, B) Teffs are obtained from naïve female T-cells from LN cells or spleen and are activated by male bone marrow-derived dendritic cells (BMDCs) + lipopolysaccharide (LPS). IgG1, DcR3.Fc, FasL/TRAIL+IgG1, and FasL/TRAIL+DcR3.Fc-treated wild-type (WT) T-cells for 24 h were subcategorized into CD8CD69 and CD8CD44 and further presented by histogram and percentage of the indicated immune cells. The cells were double stained with anti-mCD8-allophycocyanin (APC)-Cyanine7(cy7)/CD44-APC or CD69-APC. Control means without treatment, not even IgG1 alone. **(C)** Carboxyfluorescein diacetate, succinimidyl ester (CFSE) labeling assay. CFSE-labeled T-cells were treated with IgG1, DcR3.Fc, FasL/TRAIL+IgG1, or FasL/TRAIL+DcR3.Fc for 36 h before the flow cytometry anaylsis. Control means without treatment, not even IgG1 alone. **(D, E)** Regulatory T-cells were triple-stained with anti-mCD8-APC/cy7/CD25-FITC/Foxp3-Alexa Fluor and subjected to flow cytometry; the percentage of positively stained cells is indicated in each quadrant plot. Control means without treatment, not even IgG1 alone. **(F)** Detection of surface FasR in the indicated Teffs. **(G)** Dose-dependent effect of DcR3.Fc on FasR expression in the indicated Teffs. The cells were incubated with 100 ng/mL FasL, 100 ng/mL TRAIL,

and 0, 2.5, 5, 10 μg/mL DcR3.Fc, and then the FasR mRNA levels were determined using real-time PCR. **(H)** Tumor necrosis factor (TNF)-α secretion suppression. The supernatants were harvested 36 h after T-cell priming to test TNF-α using ELISA. One set of representative data from at least three experiments is shown. The Kruskal–Wallis test, followed by Bonferroni *post-hoc* analysis was used for multiple testing. **(G)** For two independent groups **(A–C, H)**, a nonparametric test, Mann–Whitney *U* test, was used. The data are presented as mean ± SE. F, FasL; I, human IgG1; D, DcR3.Fc; T, TRAIL; D0, 0 μg/mL DcR3.Fc; D2.5, 2.5 μg/mL DcR3.Fc; D5, 5 μg/mL DcR3.Fc; D10, 10 μg/mL DcR3.Fc; F0, 0 ng/mL FasL; F100, 100 ng/mL FasL; T100, 100 ng/mL TRAIL.

Results:

1. $CD8^+CD69^+$ and $CD8^+CD44^+$ cells are prominently induced with FasL+IgG1 (71.6 ± 7.3 and 65.0 ± 6.1%, respectively) and TRAIL+IgG1 (76.6 ± 2.7 and 73.0 ± 11.9%, respectively), but those cells are less induced with FasL+DcR3.Fc (65.1 ± 4.3 and 46.5 ± 10.7%, respectively) and TRAIL+DcR3.Fc (59.9 ± 3.3 and 46.5 ± 10.7%, respectively) (**Figure S9A, B**).
2. $CD8^+CD69^+$ and $CD8^+CD44^+$ cells are prominently induced with IgG1, but those cells are less induced with DcR3.Fc (**Figure S9A, B**).

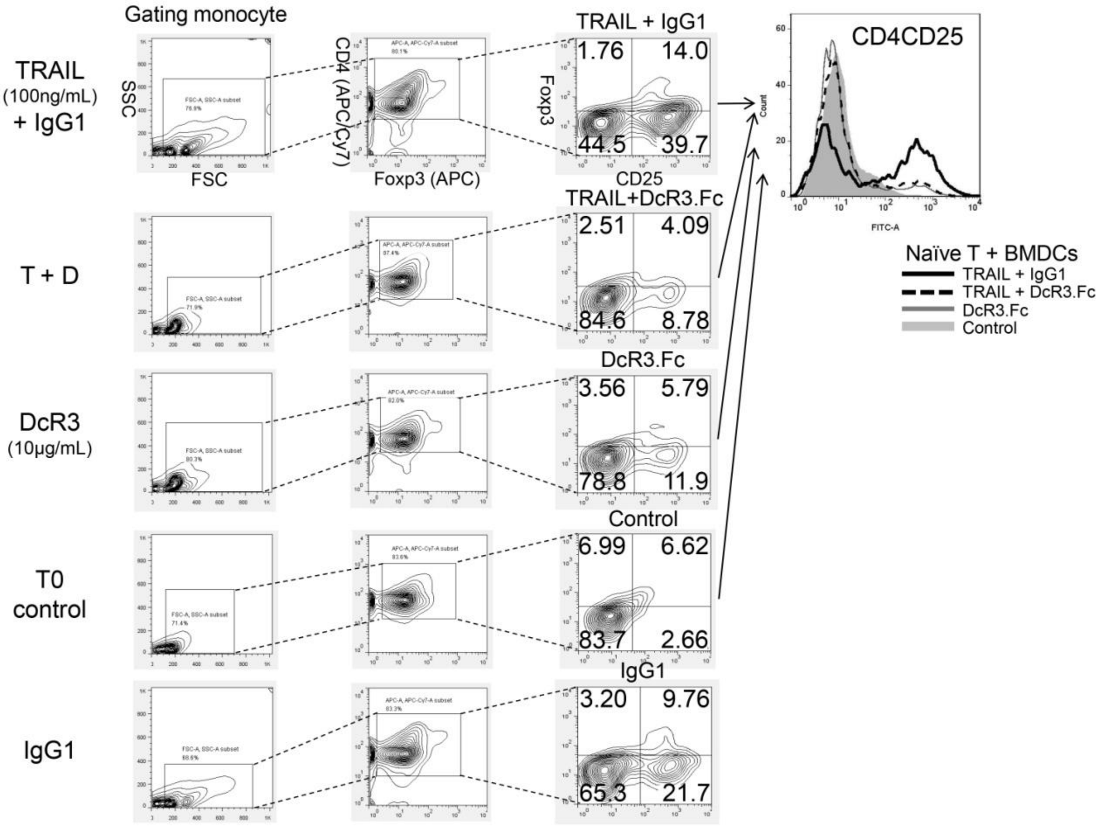

Supplementary Figure 10. Representative flow cytometry gating strategy of $CD4^{+}CD25^{+}Foxp3^{+}$ cells.

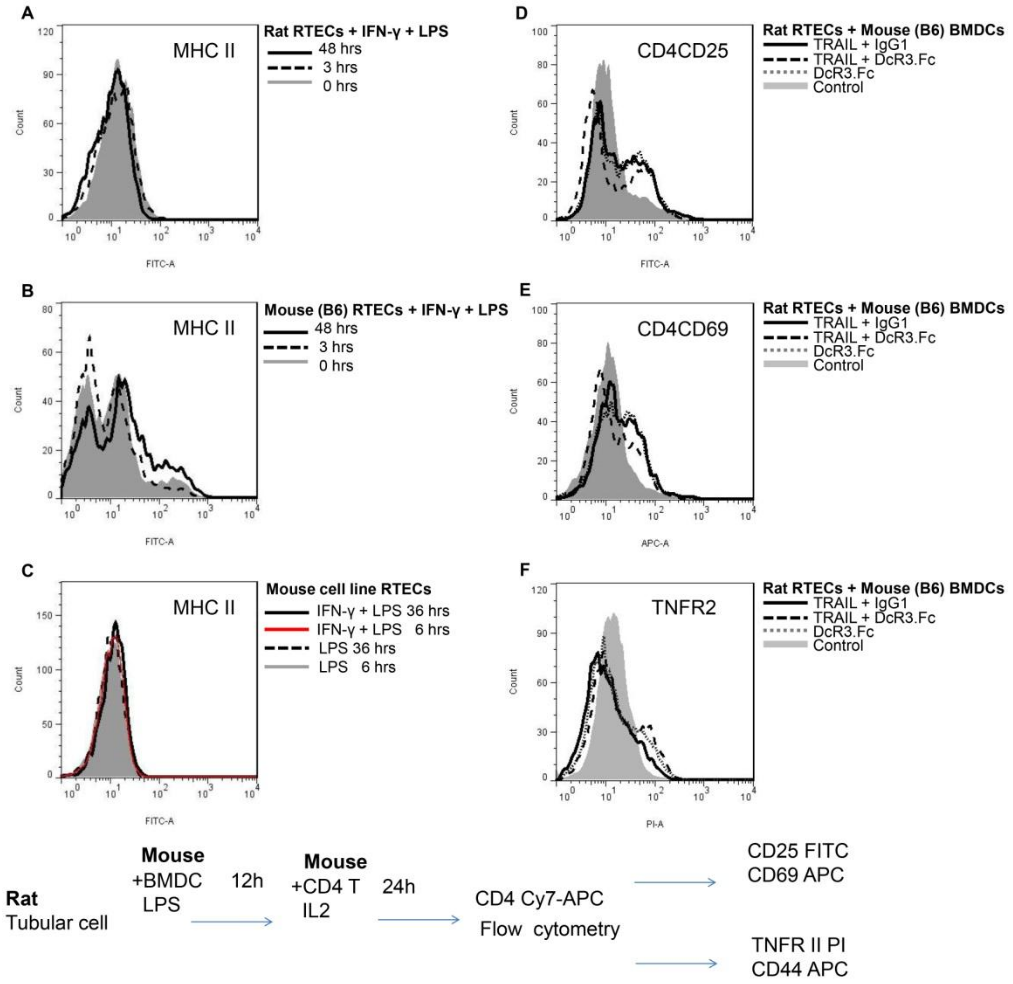

Supplementary Figure 11. MHC class II expression of mouse or rat renal tubular epithelial cells (RTECs) and proliferation of naïve CD4 T cells under major histocompatibility complex (MHC) disparity. (A) Rat (Bltw:SD) RTECs did not express class II MHC with exogenous lipopolysaccharide (LPS) and interferon-gamma (IFN-γ). **(B)** Mouse (C57BL/6) RTECs trivially expressed class II MHC with exogenous LPS and IFN-γ. **(C)** Mouse renal tubular cell line (M1) did not express class II MHC with exogenous LPS and IFN-γ. **(D, E)** DcR3.Fc slightly reduced proliferation of alloantigen-stimulated mouse $CD4^+$ T cells under MHC disparity. **(F)** DcR3.Fc slightly increased TNFR2 expression of alloantigen-stimulated mouse $CD4^+$ T cells under MHC disparity. Rat species: Bltw:SD.

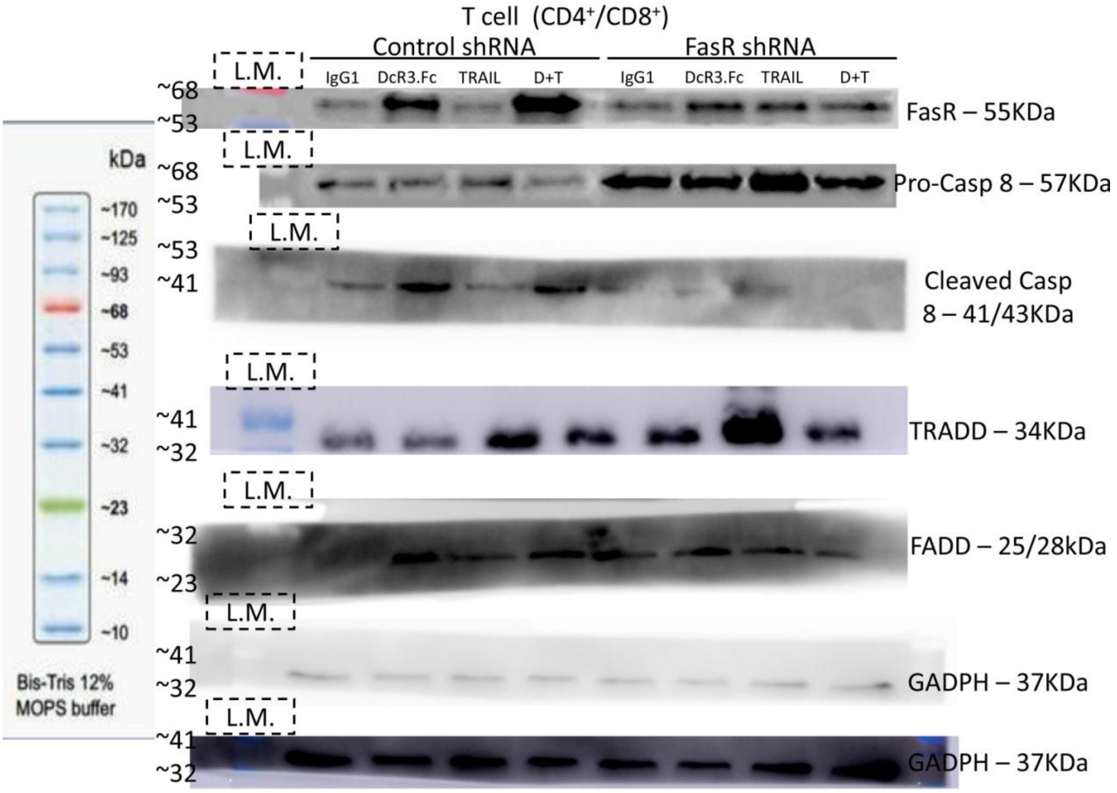

Supplementary Figure 12. Full gel pictures of immunoblotting for FasR/Fas-associated protein with death domain (FADD)/Caspase 8 pathway in T cells.

Supplementary Table 4. Baseline clinical characteristics, pathology index, and mRNA analyses among patients with or without acute T cell rejection.

	**No rejection (*n* = 12)**	**T cell rejection (*n* = 27)**	***p* value**
Clinical characteristics			
age	47.3 ± 3.9	50.3 ± 2.6	0.563
gender (male/female)	5/7	13/14	0.731
Serum creatinine (mg/dl)	2.3 ± 1.1	3.3 ± 2.4	0.201
eGFR (ml/min per 1.73m^2)			
MDRD	37.7 ± 17.7	27.1 ± 13.6	0.121
CKD-EPI	38.2 ± 18.4	26.7 ± 13.7	0.103
Banff pathology index			
tubulitis	0.0 ± 0.0	1.6 ± 1.0	<0.001
interstitial mononuclear cell infiltration	0.2 ± 0.3	2.0 ± 1.0	<0.001
Severity of rejection, *n* (%)			
mild or orderline	~	11 (40.7)	
1A & 1B	~	11 (40.7)	
2A & 2B	~	5 (18.5)	
Relative mRNA expression			
DcR3 (log10)	0.5 ± 0.3	1.7 ± 0.7	<0.001
Apoptosis genes			
Fas (log10)	0.6 ± 0.3	1.4 ± 0.7	0.004
FasL (log10)	0.5 ± 0.4	2.2 ± 0.9	<0.001
Inflammatory genes			
IL-2 (log10)	0.7 ± 0.3	2.1 ± 0.8	<0.001
TNF-α (log10)	-3.6 ± 0.6	-2.6 ± 0.7	0.002
IL-12 (log10)	-3.5 ± 1.5	-1.8 ± 0.6	0.002
IL-4 (log10)	0.6 ± 0.3	2.0 ± 0.9	<0.001
Co-stimulatory genes			
LIGHT (log10)	0.3 ± 0.2	1.2 ± 0.6	0.001
Co-inhibition genes			

CTLA-4 (log10)	- 4.2 ± 1.3	-2.8 ± 0.7	0.001
PD-1 (log10)	-4.6 ± 1.1	-2.7 ± 1.0	<0.001

We investigated kidney allograft recipients with biopsy-proved acute cellular rejection (BPACR) (n = 27) and compared those without kidney rejection (n = 12). Under the real time PCR, we found there is strong mRNA expression in DcR3, apoptotic genes (Fas, FasL), pro-inflammatory genes (IL-2, TNF-α, IL-12), anti-inflammatory gene (IL-4), co-stimulatory gene (LIGHT), and co-inhibition genes (CTLA-4, PD-1).

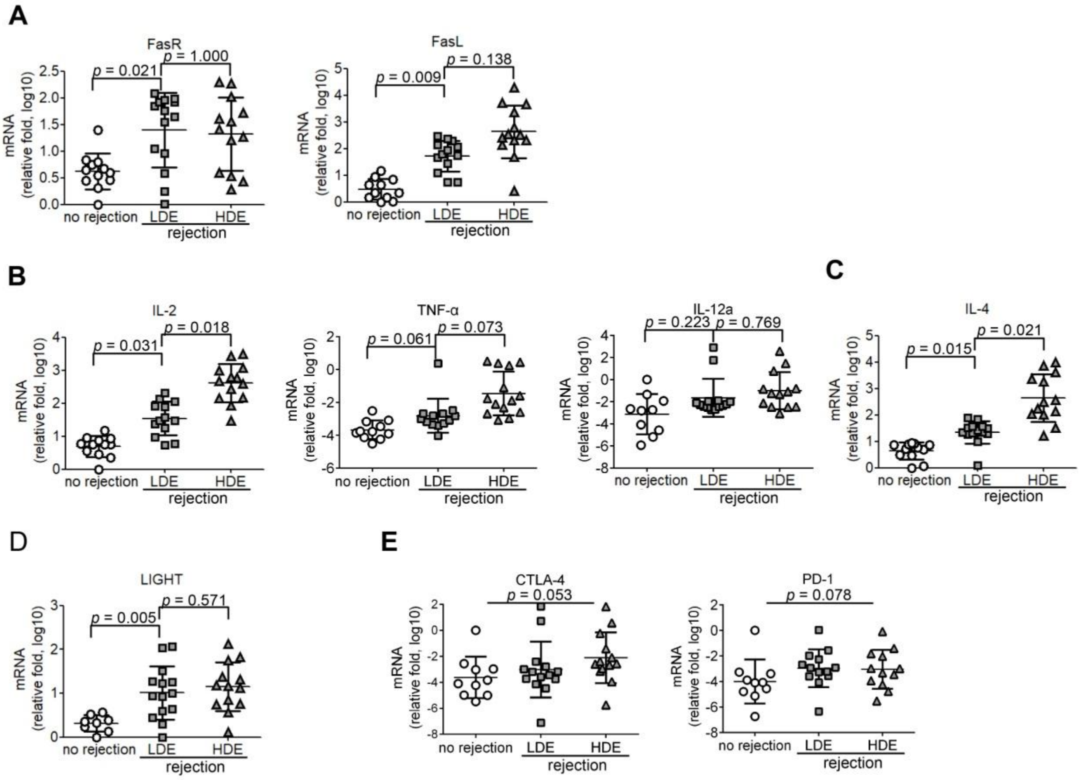

Supplementary Figure 13. mRNA analyses among patients with or without acute T cell rejection, and patients with BPACR were divided into high DcR3 expression (HDE) and low DcR3 expression (LDE). (A) apoptosis-related genes **(B)** inflammatory cytokines **(C)** anti-inflammatory cytokines **(D)** marker of effector T cells **(E)** markers of regulatory T cells. There is positive correlation among DcR3, IL-2, and IL-4. The Kruskal–Wallis test, followed by Bonferroni *post-hoc* analysis was used for multiple testing.

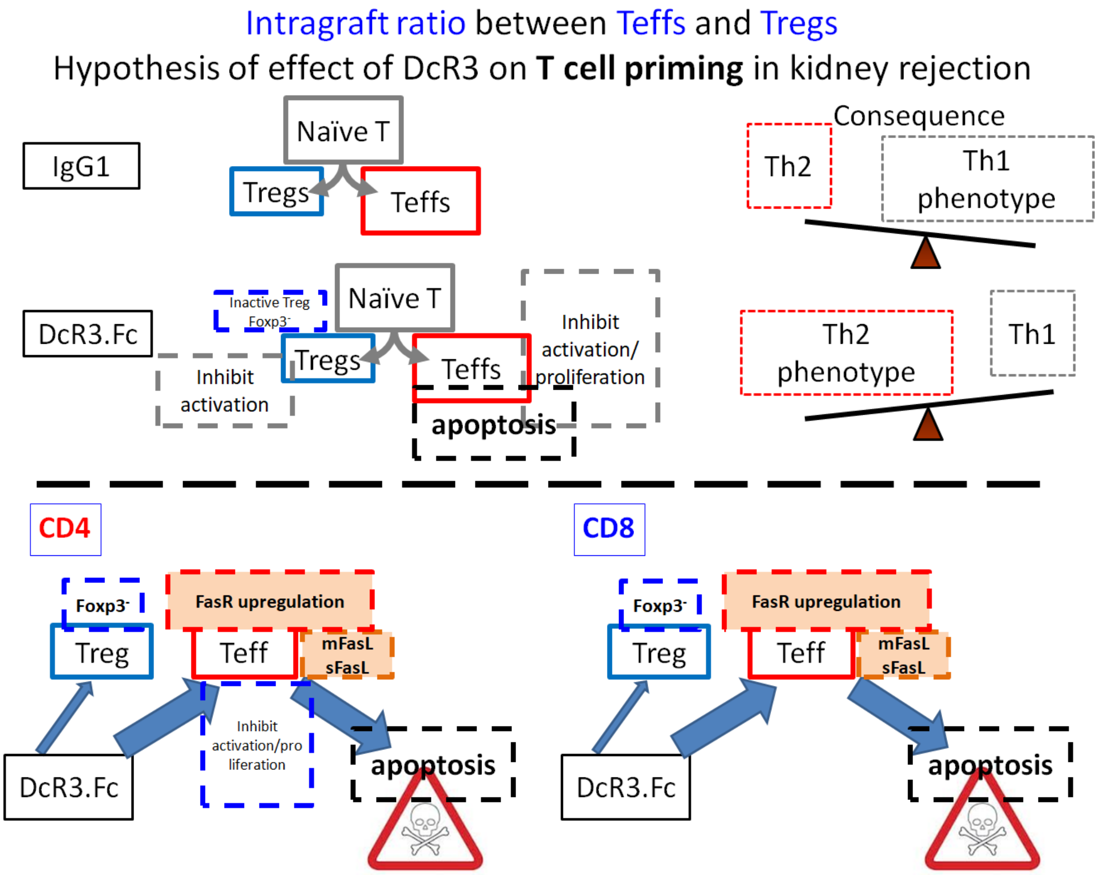

Supplementary Figure 14. Th2-like phenotype in kidney rejection after DcR3.Fc treatment.

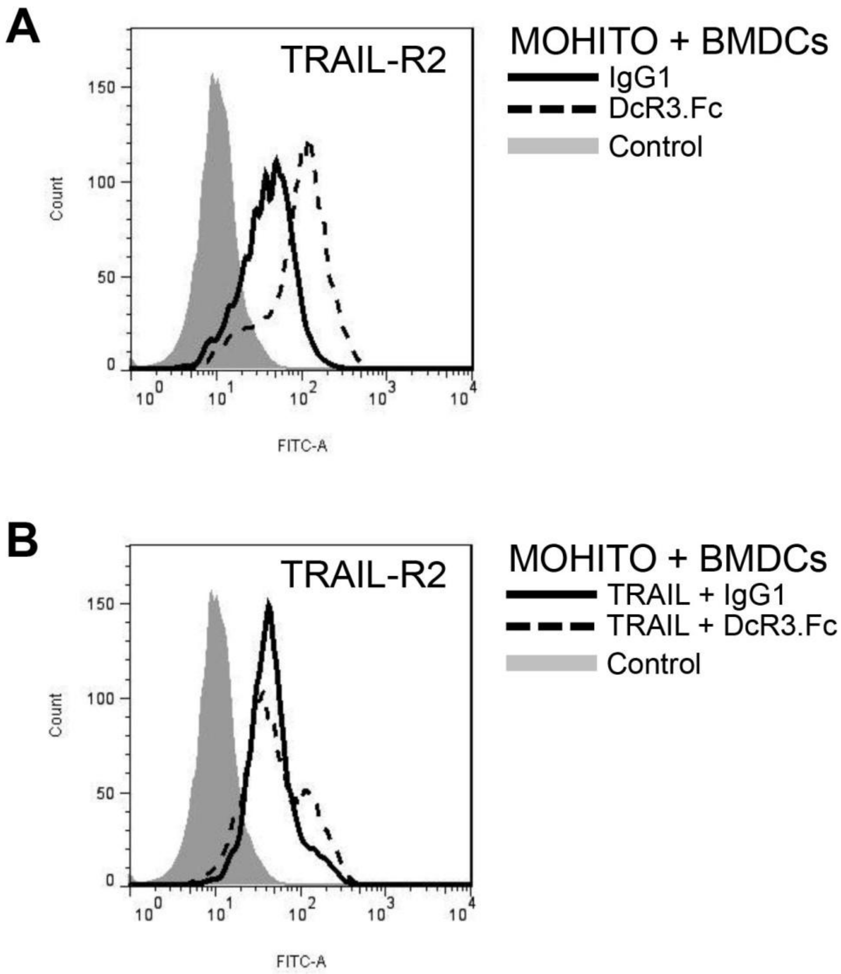

Supplementary Figure 15. TRAIL-R2 expression of the MOHITO cells after DcR3.Fc treatment.

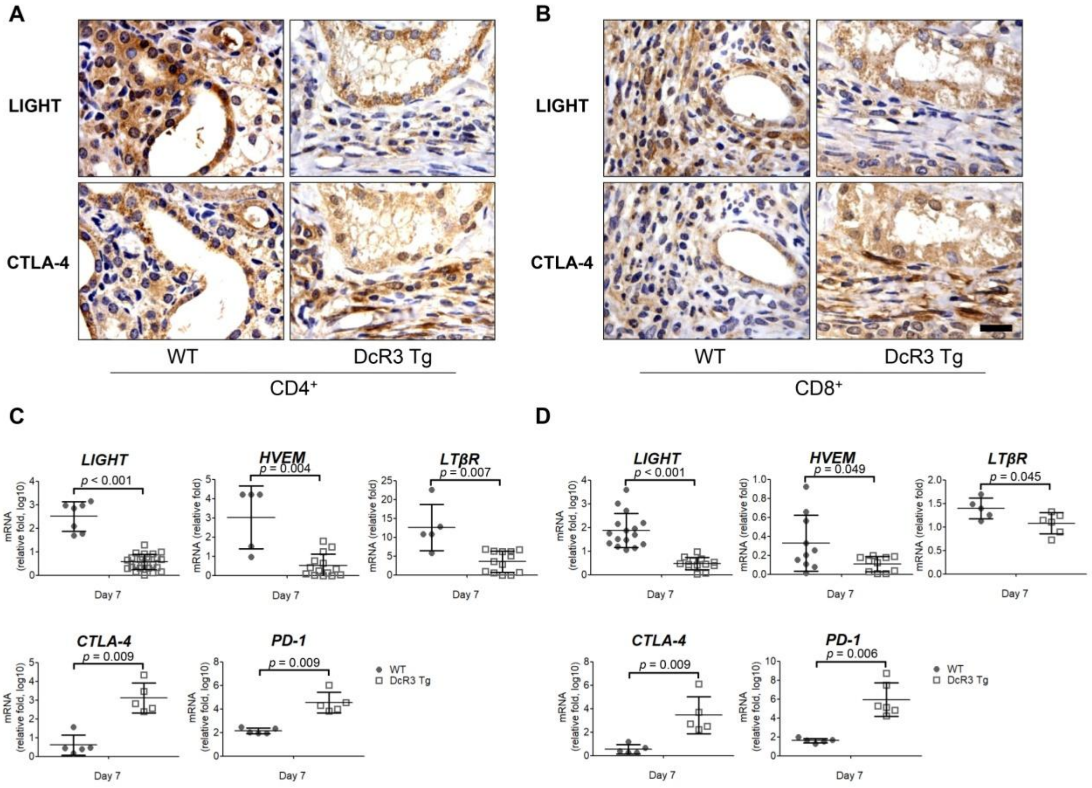

Supplementary Figure 16. Phenotypic changes of T cells between WT and DcR3 Tg mice after activated T cell injection on day 7 (*p* values were represented as a comparison between ACR WT control and ACR DcR3 Tg mice, by Mann-Whitney *U* test).
